# Metabolite profiling of rhizosphere soil of different allelopathic potential rice accessions

**DOI:** 10.1186/s12870-020-02465-6

**Published:** 2020-06-09

**Authors:** Yingzhe Li, Lining Xu, Puleng Letuma, Wenxiong Lin

**Affiliations:** 1 Fujian Provincial Key Laboratory of Agroecological Processing and Safety Monitoring, College of Life Sciences, Fujian Agriculture and Forestry University, Fuzhou, 350002 Fujian People’s Republic of China; 2 Key Laboratory of Ministry of Education for Genetics, Breeding and Multiple Utilization of Crops, College of Agriculture, Fujian Agriculture and Forestry University, Fuzhou, 350002 P. R. China; 3 Crop Science Department, National University of Lesotho, Maseru, 100 Lesotho; 4 Key Laboratory of Crop Ecology and Molecular Physiology (Fujian Agriculture and Forestry University), Fujian Province University, Fuzhou, 350002 P. R. China

**Keywords:** Fatty acids, Metabolomics, Rhizosphere, Allelopathy, Resin extraction

## Abstract

**Background:**

Identification of the allelopathy-interrelated metabolites from the allelopathic rice rhizosphere is crucial to understand the allelopathic mechanism of rice, which in turn can promote its applications to farming. In this study, the metabolites from the rhizosphere soil of five different rice lines, including allelopathic rice accession PI312777 (PI) and non-allelopathic rice accession Lemont (Le) as well as their genetic derivatives (e.g., phenylalanine ammonia-lyase (*PAL*) gene overexpression transgenic lines of PI and Le, namely, PO and LO respectively, and *PAL* RNA interference line of PI, namely, PR) were identified and comparatively analyzed to explore the positive compounds that are involved in the process of rice allelopathy.

**Results:**

The results showed that 21 non-polar compounds and 21 polar compounds differed in content in the rhizosphere soil of PI and Le, which include several volatile fatty acids and long-chain fatty acids. The relative contents of fatty acids also differed between *PAL* overexpressing or RNA interference (RNAi)-silenced line and their wild-type respectively. Acetic acid content also differed among groups, i.e., it is higher in the high allelopathic potential rice. Further analysis showed that different metabolites from the ADS8 resin-extracted phase were more abundant than that those from the ADS21 resin-extracted phase, suggesting that the allelochemicals in root exudates of allelopathic rice are mainly non-polar substances. KEGG annotation of these differential metabolites revealed that these compounds were related to nutrient metabolism, secondary metabolite synthesis, signaling substance synthesis, and toxin degradation.

**Conclusions:**

Rice allelochemicals deposited in the ADS8 resin-extracted phase were more abundant than those in the ADS21 resin-extracted phase. Allelochemicals in root exudates of allelopathic rice are mainly non-polar substances, and long-chain fatty acids are considered as allelopathy interrelated metabolites.

## Background

Allelopathy is a chemical ecological phenomenon in which donor plants release their chemical substances, called allelochemicals, into the environment through the process of secretion, volatilization, leaching, and residual degradation and affect other recipient organisms [1], which could be used to control harmful organisms, such as weeds and pests in the field. Therefore, it has become the focus of research studies [2–7]. There are two main viewpoints on the categories of allelochemicals in rice. Phenolic acids are considered as a group of allelochemicals, and the others are terpenoids and flavonoids. Rice and Chou et al. believed that phenolic acids, such as *p*-coumanic acid, ferulic acid, *p*-hydroxybenzoic acid, and oxalic acid, which are produced after the decomposition of rice residues, could be fixed by soil aggregate structure substances or humic acid and stored in the rhizosphere soil to inhibit the growth of rice seedlings and weeds [1, 8, 9]. Rimando and Seal et al. isolated allelochemicals from root extracts of rice, and they identified several major phenolic allelochemicals with inhibitory effects on the target plants [10, 11]. All of this evidence strongly supports the idea that phenolic acids are allelochemicals. However, Olofsdotter et al. were skeptical of this issue and suggested that phenolic acid compounds might not be the allelochemicals that inhibit the target weeds using 4-aminoantipyrine spectrophotometry [12]. Another argument was that phenolic acids impart inhibitory effects on the target grasses, but the dosage used for the bioassay was much higher than that detected in rhizosphere soil of allelopathic rice accession. Therefore, terpenoids, such as momilactone B, were considered as promising allelochemicals in rice because these exhibited the higher inhibitory effect on the target weeds at very lower dosages of 3–30 μmol/L [13–16]. However, all of the above results were obtained under laboratory conditions. Furthermore, the effective concentrations of phenolic acids and terpenoids used in laboratory bioassays are always higher than those of allelopathic rice released and detected in the field, which therefore has been often questioned by some scholars.

Previous studies have shown that plant allelopathy is mainly a complex rhizosphere biological process that is mediated by allelochemicals (secondary metabolites) secreted by their roots. Allelopathic plant accessions have different potentials in inhibiting target weeds, which is mainly due to differences in the functions of allelopathic genes in the synthesis of secondary metabolites and the results of the interactions between plant, soil, and rhizosphere microorganisms mediated by the allelochemicals, which in turn require a more in-depth study to elucidate the allelopathic mechanism underlying this particular rhizosphere biological process [17–20].

Previous studies have documented that several genes, including copalyl diphosphate synthase 4 (*OsCPS4*), kaurene synthase-like 4 (*OsKSL4*), and phenylalanine ammonia lyase (*OsPAL*), play roles in the process of rice allelopathy. Knocking out or silencing *OsCPS4*, *OsKSL4,* and *OsPAL* results in weaker allelopathic activity from the donor rice to the barnyardgrass. This weakened activity is attributed to reduced momilactones or phenolic acid content in root exudates [21, 22]. Among those genes, *OsPAL* is the first key gene in the phenylpropanoid metabolism and functions in the regulation of phenolic acid synthesis. Numbers of studies have documented that gene expression level of *OsPAL* from allelopathic rice accession PI312777 was higher that from non-allelopathic rice Lemont [23–26], of which these two rice accessions were widely taken as donor plant to investigate the underlying mechanism of allelopathy, PI312777 was first reported by Dilday in the field experiments to identification of allelopathic rice germplasm [27], while the Lemont rice is the contrary line with non-allelopathic activity [23–26]. Silencing of *OsPAL* gene expression in PI312777 results in a 50% reduction of weed-suppress capacity, whereas its overexpression increases the inhibition ratios of the weed by 13% [19]. The diversities of microbial community from the rhizosphere of these genetic modified rice have also changed compared to that of the rhizosphere of WT of PI312777, which are possibly correlated with changes in root exudates secretion. These metabolites are thus regarded to play crucial roles in the process of weed suppression.

Scientists have gradually realized that allelopathy is an ecogenetic trait that is involved in an extremely complex chemicobiological process in the rhizosphere ecosystem. However, more recent studies have shown that plant allelopathy includes direct allelopathic effects caused by allelochemicals, and indirect allelopathic effects mediated by allelochemicals through microbial utilization, transformation, and resynthesis. Therefore, it has been suggested that allelopathy in rice might result from the interaction of alleochemicals with specific microorganisms in rhizosphere soil [28–31]. Therefore, extensive studies have focused on assessing the interaction between allelopathic rice and rhizosphere microorganisms [19, 22, 32], including the separation and identification of allelochemicals. Thus, what other substances, besides phenolic acids and terpenoids, are allelochemicals? This requires the application of metabonomics, a bioanalytical approach that has recently emerged.

Metabolites, which are the end products of cell metabolism regulation, are the material basis of biological phenotypes, and can directly and effectively reflect biological processes and contribute to the analysis of their mechanisms [33]. Changes in metabolites, including species and quantity, are the ultimate responses of biological systems to internal or external stimulus, such as gene mutations or environmental stress [34]. Qualitative and quantitative analyses of low-molecular weight metabolites have been conducted to investigate metabolic pathways or metabolic networks, to compare and analyze the metabolic differences in macroscopic phenotypic phenomena among different biological individuals, and to study the metabolic response mechanism of substances after different induction and stress [33]. Metabolomics is usually used in combination with transcriptome and proteomics to study how changes in the physiological pathway of DNA → mRNA→protein→metabolite elicit responses to various environmental stimuli [33]. Due to the “high-dimensional and massive” nature of metabolomics data, the integration of statistical analysis of differential metabolites has facilitated accurate mining and metabolic pathway annotation, and other studies have been conducted to elucidate the mechanism underlying sample differences. Our previous studies have shown that when plant extracts or root secretions from allelopathic rice are treated with polar resin at the five-leaf stage, the inhibition rate of target weeds significantly increased, and the reverse was observed when plant extracts or root secretions were treated with a non-polar resin [35]. We also found that allelopathic rice accessions had much more microbial diversity than its counterparts because allelopathic rice synthesize and release higher amounts of allelochemicals, such as phenolic acids into rhizosphere soil environments, implying that the composition of metabolites in the rhizosphere soil ecosystem is a key factor influencing microbial community structure. In this study, allelopathic rice and non-allelopathic rice accessions as well as their genetic derivatives, namely, transgenic lines with major allelopathic gene *PAL2–1*-inhibited and *PAL2–1*-overexpressed, were used as research materials. Metabolomics was used to analyze differences in the metabolites extracted from the rhizosphere soil of different allelopathic potential rice accessions using different resins at the five-leaf rice seedling stage to improve our understanding of the chemoecological characteristics of rhizosphere soil in allelopathic rice.

## Results

### *PAL* gene and protein expression on the rice

**Fig. 1:**
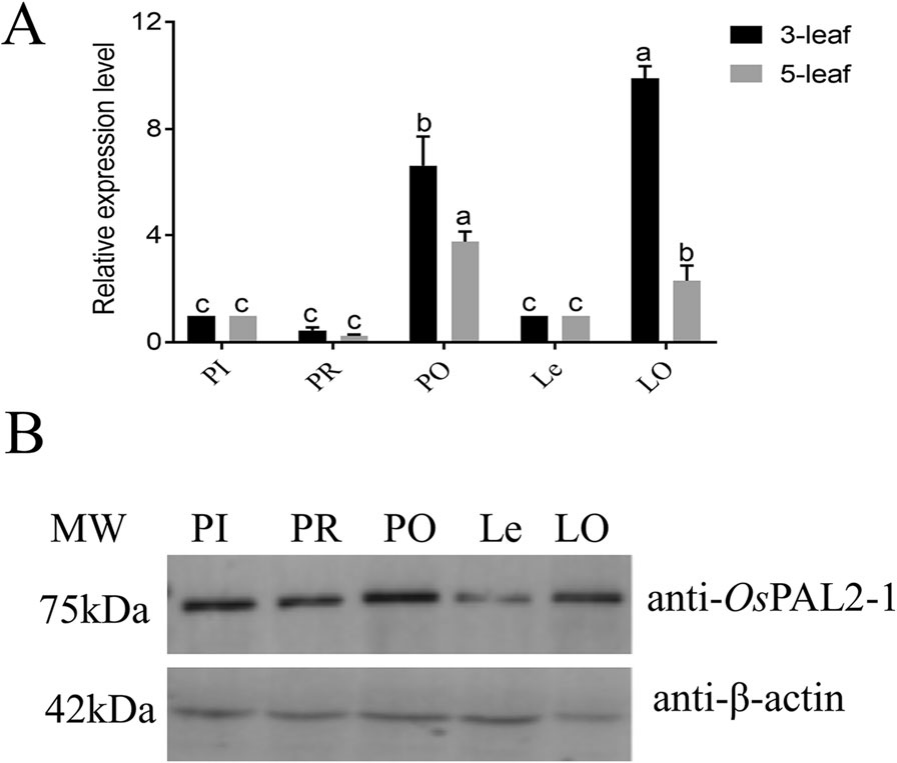
Expression of *PAL* (**a**) gene by quantitative RT-PCR (**b**) and protein expression by western blotting of rice roots. The original full-length western-blot images are shown in the Additional file 1: Figure S1

To validate the positive transgenic rice line of PI and Le, we assessed the *PAL* gene and protein expression levels in relation to the WT of PI and Le, which were higher, whereas the RNA interference transgenic line of PI showed a decrease in *PAL* gene and expression in rice relative to the WT of PI. Further comparison of the PAL protein expression on these rice lines also showed that the protein expression levels of PAL were higher in the PAL overexpression rice line than the WT, and the protein expression levels of PAL from the PAL RNAi interference of PI were lower than the WT of PI (Fig. 1). In addition, the changes in *PAL* expression in rice did not substantially influence the phenotypes and growth period traits.

### Principal component analysis (PCA) of different polar metabolites from rice rhizosphere soil

PCA was performed on the samples to assess the variability of each sample within the group. For the non-polar metabolites from rhizosphere soil of PI, PR, PO, Le, and LO, and those metabolites from the same soil without rice plants (blank soil), the results showed that six groups of samples were distinctly clustered, indicating that the metabolites from different rice rhizospheric soil were clearly different, whereas the three repeats from the same group only showed slight differences (Additional file 2). Specifically, metabolites from the samples of PI and Le were deposited in the first principal component (t [1]), whereas PI was distinguished from Le and similar to the blank control CK (Additional file 2). For the three transformed rice lines, PO was distinguished from PR and LO among transgenic lines using principal component II (t [2]).

PCA was also performed on six samples extracted from the ADS-21 values. The results showed that although the samples of PO, Le, and LO were roughly close to each other, these were well separated among samples of the six groups, indicating that the rhizosphere soil samples from different rice accessions could be clearly distinguished, but the samples within the same group also exhibited slight differences (Additional file 3). The samples from the PR and PO transgenic lines and Le non-allelopathic rice were separated from the sample of PI allelopathic rice, Le was isolated from its counterpart, LO, and PI was distinguished well from Le by principal component I (t [1]). Furthermore, the samples of PO and LO were closer to those of Le, especially PO and Le samples (Additional file 3).

### Metabolites in the ADS8 and ADS21 resin-extracted phase

ADS8 and ADS21 resins mainly adsorb non-polar and polar compounds, respectively, compared to the metabolites from the rhizosphere soil, which were respectively deposited onto the ADS8 resin and ADS21 resin, showing that the compounds from ADS8 resin, which were classified as non-polar compounds, were more abundant than the compounds from the ADS21 resin, which were polar compounds. Among the five rice lines, there were relatively higher quantity and abundance of non-polar substances, such as beta.-n-butylether, 9-octadecenoic acid, and pentadecane, than polar substances, such as 2-butanone, d-mannitol, and nitrile. Acetic acid was detected in the rhizosphere soil of PI, Le, PR, PO, and LO, using the ADS8 resin. Based on the ADS21 resin-extracted phase, d-glucose, propenal, glutaric acid, and polar compounds were detected in the rhizosphere soil of PI, Le, PR, PO, and LO. The results indicate that rhizosphere soil metabolites with non-polar properties were more abundant than those with polar properties.

### Changes in ADS8 resin-extracted fatty acids from the rhizosphere soil of rice

Previous studies have mainly focused on changes and differences among phenolic acids and terpenoids, two kinds of allelochemicals from different allelopathic rice accessions. Furthermore, the fatty acids, e.g., long-chain fatty acids and volatile fatty acids, were also regarded to play potential roles in allelopathy [1, 36, 37]. In the ADS8 resin-extracted phase from the rhizosphere soil of allelopathic rice PI and Le, eight kinds of fatty acids were detected at different concentrations in the two samples, with acetic acid, pentanoic acid, and heptanoic acid showing higher relative contents from PI than Le, whereas cyclopropanecarboxylic acid, methylphosphonic acid, octanoic acid, and fumaric acid exhibited higher concentrations in the rhizosphere soil of Le. Silencing *PAL* gene expression in PI rendered levels of hexanoic acid, acetic acid, heptanoic acid, and pentanoic acid, lower than in WT PI. It also rendered the concentration of hexanedioic acid higher than in WT PI. When the *PAL* gene was overexpressed in PI, the levels of five fatty acids, including benzoic acid, propenoic acid, cyclopropanecarboxylic acid, and azelaic acid, increased and levels of pentanoic acid and thioacetic acid decreased. Changes in fatty acid levels were also detected in the *PAL* overexpressing line of Le. This showed that butanoic acid and acetic acid levels were higher in the LO than in the WT Le. In addition, there were eight fatty acids, namely, 9-octadecenoic acid, heptanoic acid, cyclohexanecarboxylic acid, 3-amino-2,3-dihydrobenzoic acid, succinic acid, 2,2-bis(4-hydroxyphenyl)-propanoic acid, octanoic acid, and decanoic acid which exhibited lower concentrations in the LO compared to Le (Table 1).

**Table 1:** Comprehensive comparison of ADS8 resin extracted fatty acids from the rhizosphere soil of rice

VIP	Name	PI:Le	VIP	Name	PI:PR	VIP	Name	PO:PI	VIP	Name	LO:Le
1.90713	Acetic acid	U	2.70187	Hexanoic acid	U	1.25548	Benzoic acid	U	1.70646	Butanoic acid	U
1.59614	Pentanoic acid	U	1.67186	Acetic acid	U	1.25174	Propenoic acid	U	1.57475	Acetic acid	U
1.27283	Heptanoic acid	U	1.11581	Heptanoic acid	U	1.18536	Cyclopropanecarboxylic acid	U	2.48717	9-Octadecenoic acid	D
1.30204	Cyclopropanecarboxylic acid	D	1.25499	Pentanoic acid	U	1.09064	Azelaic acid	U	1.68056	Heptanoic acid	D
1.24085	Methylphosphonic acid	D	1.36746	Hexanedioic acid	D	1.47045	Pentanoic acid	D	1.49533	Cyclohexanecarboxylic acid	D
1.17	Octanoic acid	D				1.37916	Thioacetic acid	D	1.48714	3-Amino-2,3-dihydrobenzoic acid	D
1.0572	Fumaric acid	D							1.44648	Succinic acid	D
1.20765	8,11,14-Eicosatrienoic acid	U							1.14348	2,2-Bis(4-hydroxyphenyl)-propanoic acid	D
									1.11278	Octanoic acid	D
									1.08017	Decanoic acid	D

Note: *U* upregulated expression. *D* downregulated expression

A comprehensive comparison among the soil samples from PI and Le, PI and PR, PO and PI, as well as LO and Le indicated that acetic acid is one of the potential compounds from the non-polar phase that is involved in the allelopathic process.

### Changes in ADS21 resin-extracted fatty acids from the rhizosphere soil of rice

Identification of the ADS21 resin-extracted phase showed that several fatty acids were also deposited in this resin. Comparison of fatty acids deposited in the resins, which were used for the extraction of PI and Le rhizosphere soil showed that there were seven fatty acids occurring at different concentrations in the two rice samples. Among these seven compounds, heptanedioic acid, hexanoic acid, and pentanedioic acid showed relatively higher levels in PI than Le, whereas 8,11,14-eicosatrienoic acid, 9-octadecanoic acid, hexadecanoic acid and dodecanoic acid exhibited relatively lower concentrations in PI than Le. In the rhizosphere soil of the PR rice line, four fatty acids showed differential concentrations in the PI and PR, of which pentanedioic acid and cyclopropanecarboxylic acid were higher in the PI than PR, but the concentrations of tetradecanoic acid and 10-undecenoic acid were lower in PI than PR. When *PAL* was overexpressed in the PI, the 1,2-benzenedicarboxylic acid, cyclohexanecarboxylic acid, and hexanoic acid exhibited higher levels of accumulation in PO than PI, and the concentration of cyclopropanecarboxylic acid in these two rice samples showed the opposite trend. In addition, there were eight fatty acids that were differentially generated in Le than in its transgenic line LO. The concentrations of isobutyric acid, cis-2-dodecenoic acid, hexadecanoic acid, decanedioic acid, and cyclopropanecarboxylic acid were higher in LO than in Le, whereas that of 9-octadecanoic acid, 2-butenoic acid, and dodecanoic acid were lower in the soil sample of LO than Le (Table 2).

**Table 2:** Comprehensive comparison of ADS21 resin extracted fatty acids from the rhizosphere soil of rice

VIP	Name	PI:Le	VIP	Name	PI:PR	VIP	Name	PO:PI	VIP	Name	LO:Le
1.95187	Heptanedioic acid	U	1.48364	Pentanedioic acid	U	2.85069	1,2-Benzenedicarboxylic acid	U	2.19198	Isobutyric acid	U
1.71039	Hexanoic acid	U	1.14989	Cyclopropanecarboxylic acid	U	1.28803	Cyclohexanecarboxylic acid	U	1.6433	cis-2-Dodecenoic acid	U
1.63059	Pentanedioic acid	U	1.55034	Tetradecanoic acid	D	1.17727	Hexanoic acid	U	1.4779	Hexadecanoic acid	U
2.0113	8,11,14-Eicosatrienoic acid	D	1.50193	10-Undecenoic acid	D	1.33051	Cyclopropanecarboxylic acid	D	1.43455	Decanedioic acid	U
1.7027	9-octadecanoic acid	D							1.05361	Cyclopropanecarboxylic acid	U
1.58059	Hexadecanoic acid	D							1.04824	Benzoic acid	U
1.06824	Dodecanoic acid	D							2.27918	9-octadecanoic acid	D
									2.07028	Carbamic acid	D
									1.94407	m-Cresotic acid	D
									1.20318	2-Butenoic acid	D
									1.09957	Dodecanoic acid	D

Note: *U* upregulated expression. *D* downregulated expression

Among the above results, hexanoic acid and pentanedioic acid from the polar phase exhibited higher levels of accumulation in the rhizosphere soil of high allelopathic potential rice, thereby suggesting their potential role in rice allelopathic inhibition of weeds.

### Differences in metabolites from the rhizosphere soils of PI and Le

**Fig. 2:**
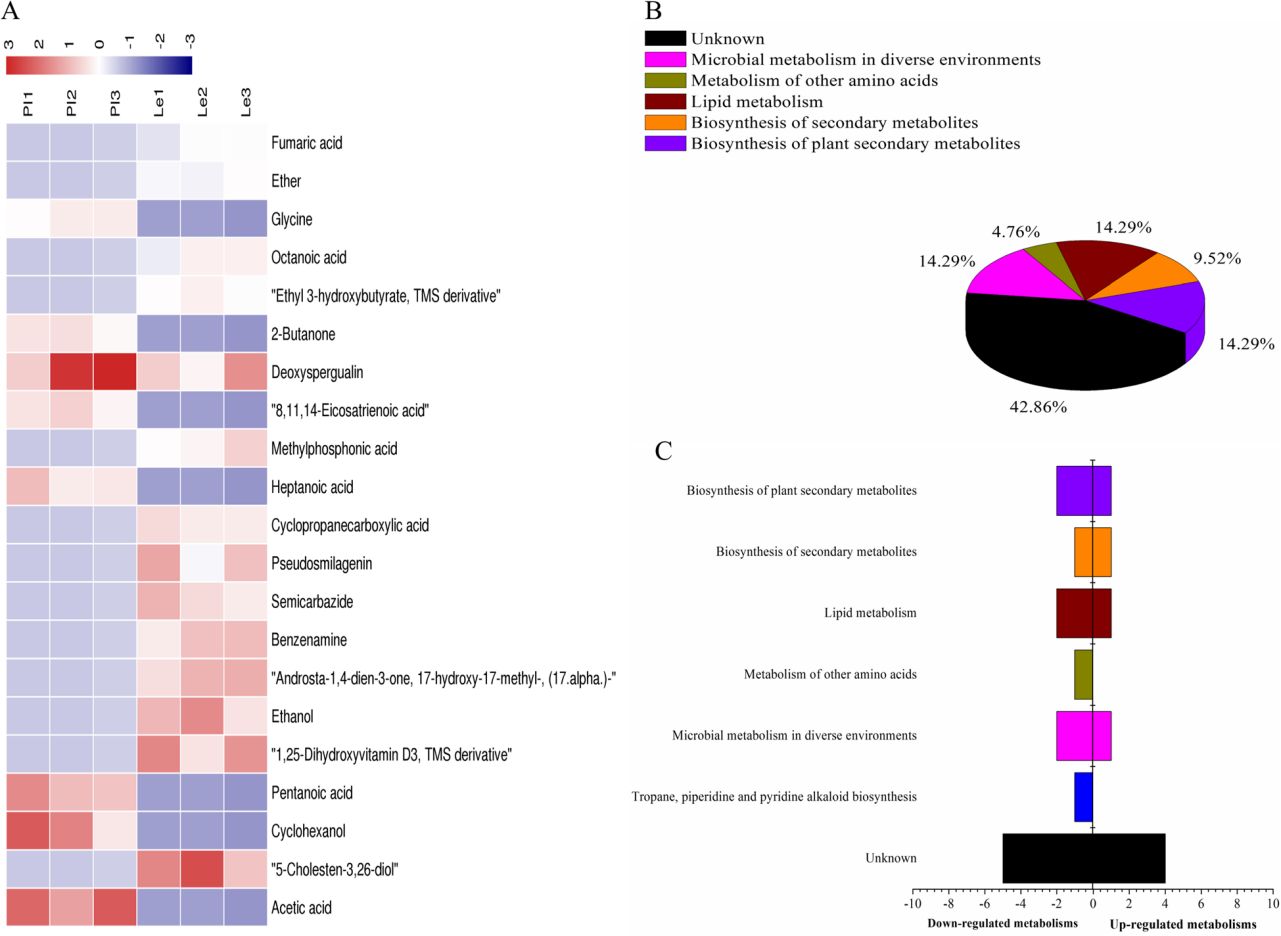
Analysis of differential metabolites between PI and Le samples absorbed by ADS-8 resin from rhizosphere soil. **a** The relative content of the metabolites between PI and Le displayed in the heat map, and the differential substances are arranged according to the VIP values from small to large. Each row represents a compound: of which the value of each compound represents the relative content directly normalized on the scale of the graph. Bluish color represents low content while reddish color represents high content. **b** The possible metabolic pathway distribution of single metabolic substance between PI and Le. **c** The left side represents the number of single differential substance in the same pathway where PI is lower than Le, and the right side is the opposite

A full comparison of the ADS8-extracted phase of rhizospheric soil of PI and Le showed that there were mainly 21 compounds that were differentially expressed across these two samples (Fig. 2, Table 3), and 8 compounds—acetic acid, cyclohexanol, pentanoic acid, heptanoic acid, 8,11,14-eicosatrienoic acid, deoxyspergualin, 2-butanone, and glycine, more abundant in the extraction from rhizosphere soil of PI than Le. Acetic acid showed the most pronounced difference (VIP = 1.90713). In contrast, there were 13 compounds that showed lower content in the PI rhizosphere soil than the Le rhizosphere soil, which include 5-cholesten-3,26-diol, 1,25-dihydroxyvitamin D3, ethanol, androsta-1,4-dien-3-one, 17-hydroxy-17-methyl-, benzenamine, semicarbazide, pseudosmilagenin, cyclopropanecarboxylic acid, methylphosphonic acid, ethyl 3-hydroxybutyrate, ether, and fumaric acid (Fig. 2a and b). These compounds participated in microbial metabolism in diverse environments (acetate, glycine, fumarate, aniline, ethanol, cyclohexanol, and cyclopropanecarboxylate), endocrine (calcitriol), and other factor-regulated calcium reabsorption, and biosynthesis of fatty acids (octanoic acid) and unsaturated fatty acids [(8Z,11Z,14Z)-icosatrienoic acid involved].

**Table 3:** The biomarkers between PI and Le samples absorbed by ADS-8 resin from rhizosphere soil

Name	KEGG	VIP	PI	Le	Participating in metabolic pathways
Acetic acid	C00033	1.90713	U	D	ko01120 Microbial metabolism in diverse environments (7) cpd:C00033 Acetate cpd:C00037 Glycine cpd:C00122 Fumarate cpd:C00292 Aniline cpd:C00469 Ethanol cpd:C00854 Cyclohexanol cpd:C16267 Cyclopropanecarboxylate
5-Cholesten-3,26-diol	C17336	1.71897	D	U	
Cyclohexanol	C00854	1.63707	U	D	
Pentanoic acid	C00803	1.59614	U	D	
1,25-Dihydroxyvitamin D3, TMS derivative	C01673	1.58096	D	U	
Ethanol	C00469	1.50588	D	U	
Androsta-1,4-dien-3-one, 17-hydroxy-17-methyl-, (17. alpha.)-	D00389	1.49721	D	U	
Benzenamine	C00292	1.4247	D	U	
Semicarbazide	C02077	1.39372	D	U	ko04961 Endocrine and other factor-regulated calcium reabsorption (1) cpd:C01673 Calcitriol
Pseudosmilagenin	C19650	1.37739	D	U	
Cyclopropanecarboxylic acid	C16267	1.30204	D	U	ko01040 Biosynthesis of unsaturated fatty acids (1) cpd:C03242 (8Z,11Z,14Z)-Icosatrienoic acid
Heptanoic acid	C17714	1.27283	U	D	
Methylphosphonic acid	C20396	1.24085	D	U	ko00061 Fatty acid biosynthesis (1) cpd:C06423 Octanoic acid
8,11,14-Eicosatrienoic acid	C03242	1.20765	U	D	
Deoxyspergualin	D08032	1.18815	U	D	
2-Butanone	C02845	1.17795	U	D	
Ethyl 3-hydroxybutyrate, TMS derivative	C03499	1.17133	D	U	
Octanoic acid	C06423	1.17	D	U	
Glycine	C00037	1.09679	U	D	
Ether	C13240	1.09528	D	U	
Fumaric acid	C00122	1.0572	D	U	

Note: *U* upregulated expression. *D* downregulated expression

**Fig. 3:**
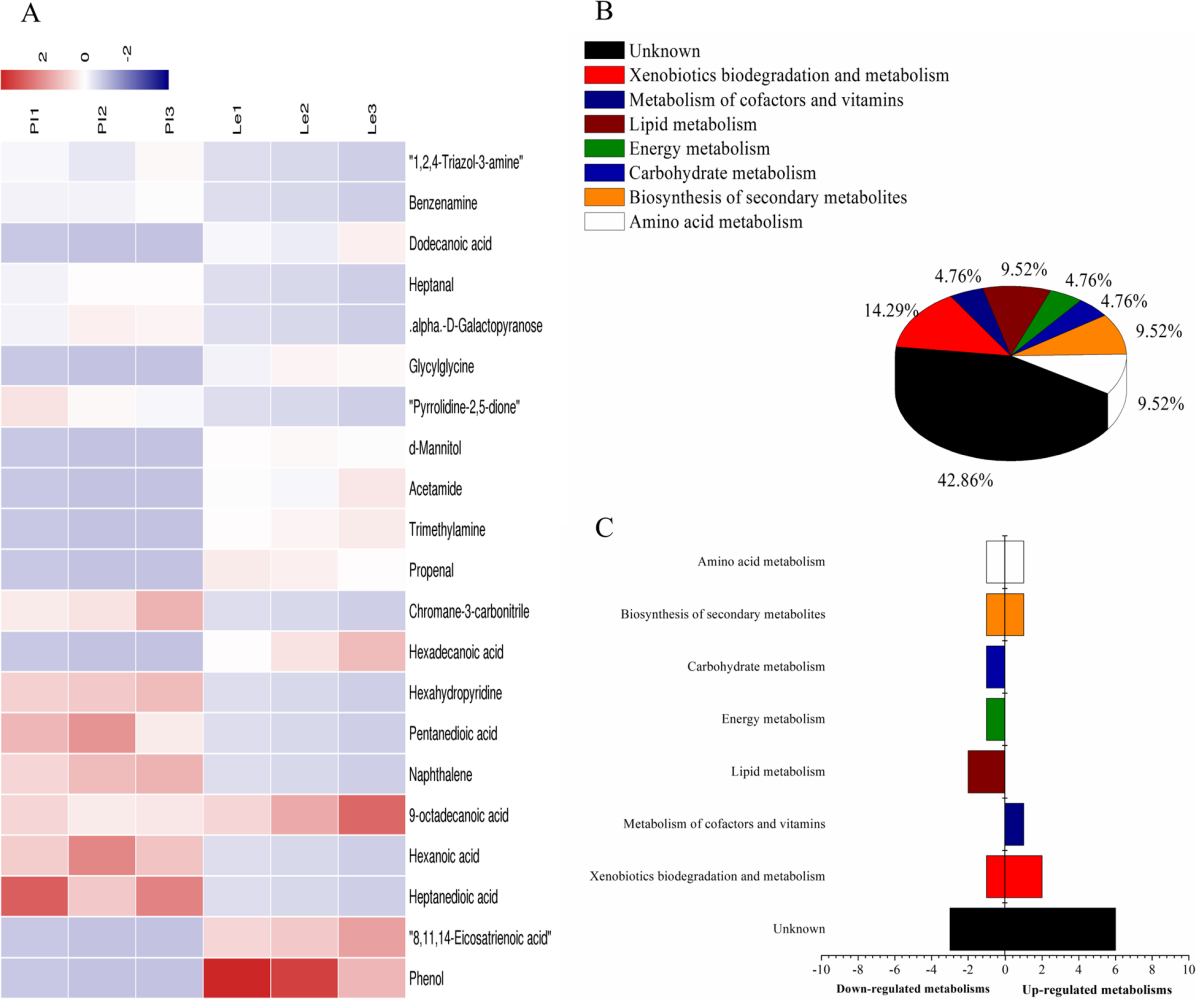
Analysis of differential metabolites between PI and Le samples absorbed by ADS-21 resin from rhizosphere soil. **a** The relative content of the metabolites between PI and Le is shown in the heat map, and the differential substances are arranged according to the VIP values from small to large. Each row represents a compound. The value of each compound represents the relative content directly normalized on the scale of the graph. Blue represents low content while red represents high content. **b** The possible metabolic pathway distribution of single metabolic substance between PI and Le. **c** The left side represents the number of single differentia substance in the same pathway where PI is lower than Le, and the right side is the opposite

Identification and comparative analysis of the ADS21-extracted phase also showed that there were 21 compounds that were differentially expressed in the rhizosphere soil of PI and Le. Among the 21 compounds, 11 compounds were relatively higher in the PI than Le, whereas 10 other compounds were higher in the Le. Phenol was the most significantly differentially expressed metabolite (VIP = 2.74924), which was higher in Le than PI. The differential content compounds between these two rice lines might participate in the metabolic pathways (four compounds, namely, hexadecanoic acid, naphthalene, pimelate and (8Z,11Z,14Z)-icosatrienoic acid, involved on the pathway), aminobenzoate degradation (phenol and aniline involved), drug metabolism-cytochrome P450 (acrolein involved), lysine degradation (glutarate involved), and ABC transporters (mannitol involved) (Fig. 3a and b, Table 4).

**Table 4:** The biomarkers between PI and Le samples absorbed by ADS-21 resin in rhizosphere soil

Name	KEGG	VIP	PI	Le	Participating in metabolic pathways
Phenol	C00146	2.74924	D	U	ko01100 Metabolic pathways (4) cpd:C00249 Hexadecanoic acid cpd:C00829 Naphthalene cpd:C02656 Pimelate cpd:C03242 (8Z,11Z,14Z)-Icosatrienoic acid
8,11,14-Eicosatrienoic acid	C03242	2.0113	D	U	
Heptanedioic acid	C02656	1.95187	U	D	
Hexanoic acid	C01585	1.71039	U	D	
9-octadecanoic acid	C01530	1.7027	D	U	
Naphthalene	C00829	1.6561	U	D	ko00627 Aminobenzoate degradation (2) cpd:C00146 Phenol cpd:C00292 Aniline
Pentanedioic acid	C00489	1.63059	U	D	
Hexahydropyridine	C01746	1.61662	U	D	
Hexadecanoic acid	C00249	1.58059	D	U	ko00982 Drug metabolism - cytochrome P450 (1) cpd:C01471 Acrolein
Chromane-3-carbonitrile	C11697	1.47238	U	D	
Propenal	C01471	1.39017	D	U	ko00310 Lysine degradation (1) cpd:C00489 Glutarate
Trimethylamine	C00565	1.37453	D	U	
Acetamide	C06244	1.24142	D	U	ko02010 ABC transporters (1) cpd:C00392 Mannitol
d-Mannitol	C00392	1.21903	D	U	
Pyrrolidine-2,5-dione	C07273	1.18807	U	D	
Glycylglycine	C02037	1.13566	D	U	
.alpha.-D-Galactopyranose	C00738	1.1085	U	D	
Heptanal	C14390	1.08526	U	D	
Dodecanoic acid	C02679	1.06824	D	U	
Benzenamine	C00292	1.01478	U	D	
1,2,4-Triazol-3-amine	C11261	1.0102	U	D	

Note: *U* upregulated expression. *D* downregulated expression

The results of the analysis of the metabolic pathways in which each of the 21 differential metabolites were intricate, as shown in Fig. 3c and d, illustrated that all of the differential metabolites involved in xenobiotics biodegradation and metabolism and metabolism of cofactors and vitamins were higher in content in PI than in Le. In contrast, the differentially expressed metabolites involved in lipid metabolism, energy metabolism and carbohydrate metabolism were downregulated in PI.

### Differences in the expression of metabolites from the rhizosphere soils of PI, PR, and PO

**Fig. 4:**
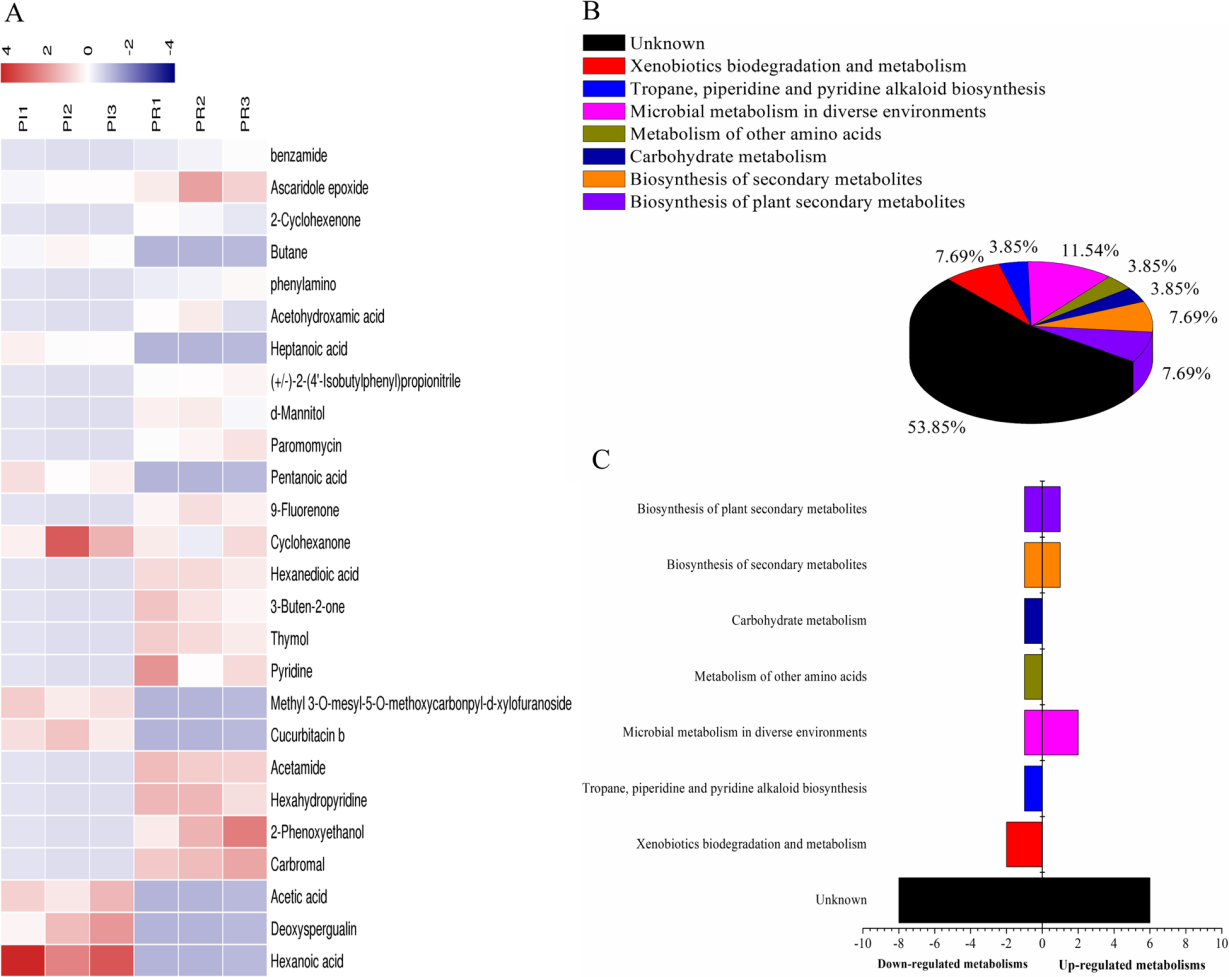
Analysis of differential metabolites between PI and PR samples absorbed by ADS-8 resin from rhizosphere soil. **a** The relative content of the metabolites between PI and PR is presented in the heat map, and the differential substances are arranged according to the VIP values from small to large. Each row represents a compound. The value of each compound represents the relative content directly normalized on the scale of the graph. Blue represents low content while red represents high content; **b** The possible metabolic pathway distribution of single metabolic substance between PI and PR. **c** The left side represents the number of single differential substance in the same pathway where PI is lower than PR, and the right side is the opposite

The samples from the rhizosphere soil of PI and PR were analyzed according to the above methods, and the results indicated that there are 26 key differential metabolites between PI and PR samples (Fig. 4a and b, Table 5), of which hexanoic acid was significantly different. All of these might be involved in the metabolic pathways: Microbial metabolism in diverse environments (six substances are involved in the process, i.e., acetate, L-aspartate, cyclohexanone, adipate, fluoren-9-oneand benzamide), tropane, piperidine, and pyridine alkaloid biosynthesis (piperidine involved in the pathway), fructose and mannose metabolism (mannitol takes part in the process) and neomycin, kanamycin, and gentamicin biosynthesis (paromomycin involved).

**Table 5:** The biomarkers between PI and PR samples absorbed by ADS-8 resin in rhizosphere soils

Name	KEGG	VIP	PI	PR	Participating in metabolic pathways
Hexanoic acid	C01585	2.70187	U	D	ko01120 Microbial metabolism in diverse environments (6) cpd:C00033 Acetate cpd:C00049 L-Aspartate cpd:C00414 Cyclohexanone cpd:C06104 Adipate cpd:C06712 Fluoren-9-one cpd:C09815 Benzamide
Deoxyspergualin	D08032	1.69418	U	D	
Acetic acid	C00033	1.67186	U	D	
Carbromal	D02619	1.62782	D	U	
2-Phenoxyethanol	D08359	1.59034	D	U	
Hexahydropyridine	C01746	1.55401	D	U	
Acetamide	C06244	1.54784	D	U	
Cucurbitacin b	C08794	1.54166	U	D	ko00960 Tropane, piperidine and pyridine alkaloid biosynthesis (1) cpd:C01746 Piperidine
Methyl 3-O-mesyl-5-O -methoxycarbonpyl-d-xylofuranoside	C00049	1.52048	U	D	
Pyridine	C00747	1.41385	D	U	ko00051 Fructose and mannose metabolism (1) cpd:C00392 Mannitol
Thymol	C09908	1.4007	D	U	
3-Buten-2-one	C20701	1.37027	D	U	ko00524 Neomycin, kanamycin and gentamicin biosynthesis (1) cpd:C00832 Paromomycin
Hexanedioic acid	C06104	1.36746	D	U	
Cyclohexanone	C00414	1.3182	U	D	
9-Fluorenone	C06712	1.27628	D	U	
Pentanoic acid	C00803	1.25499	U	D	
Paromomycin	C00832	1.20995	D	U	
d-Mannitol	C00392	1.20081	D	U	
(+/−)-2-(4′-Isobutylphenyl) propionitrile	C04469	1.13964	D	U	
Heptanoic acid	C17714	1.11581	U	D	
Acetohydroxamic acid	C06808	1.04682	D	U	
phenylamino	C01302	1.03838	D	U	
Butane	D03186	1.034	U	D	
2-Cyclohexenone	C02395	1.03148	D	U	
Ascaridole epoxide	C09836	1.01456	U	D	
benzamide	C09815	1.00777	D	U	

Note: *U* upregulated expression. *D* downregulated expression

The relative content of 26 kinds of differential metabolites across PI and PR were analyzed, and possible metabolic pathways were established for each substance involved (Fig. 4c and d). Results showed that the levels of acids, such as hexanoic acid, acetic acid, and heptanoic acid were higher in PI than in PR. The mainly metabolic pathway that these differential metabolites involved were microbial metabolism in diverse environments. Substances involved in microbial metabolism in diverse environments and biosynthesis of secondary metabolites were up-regulated in PI.

**Fig. 5:**
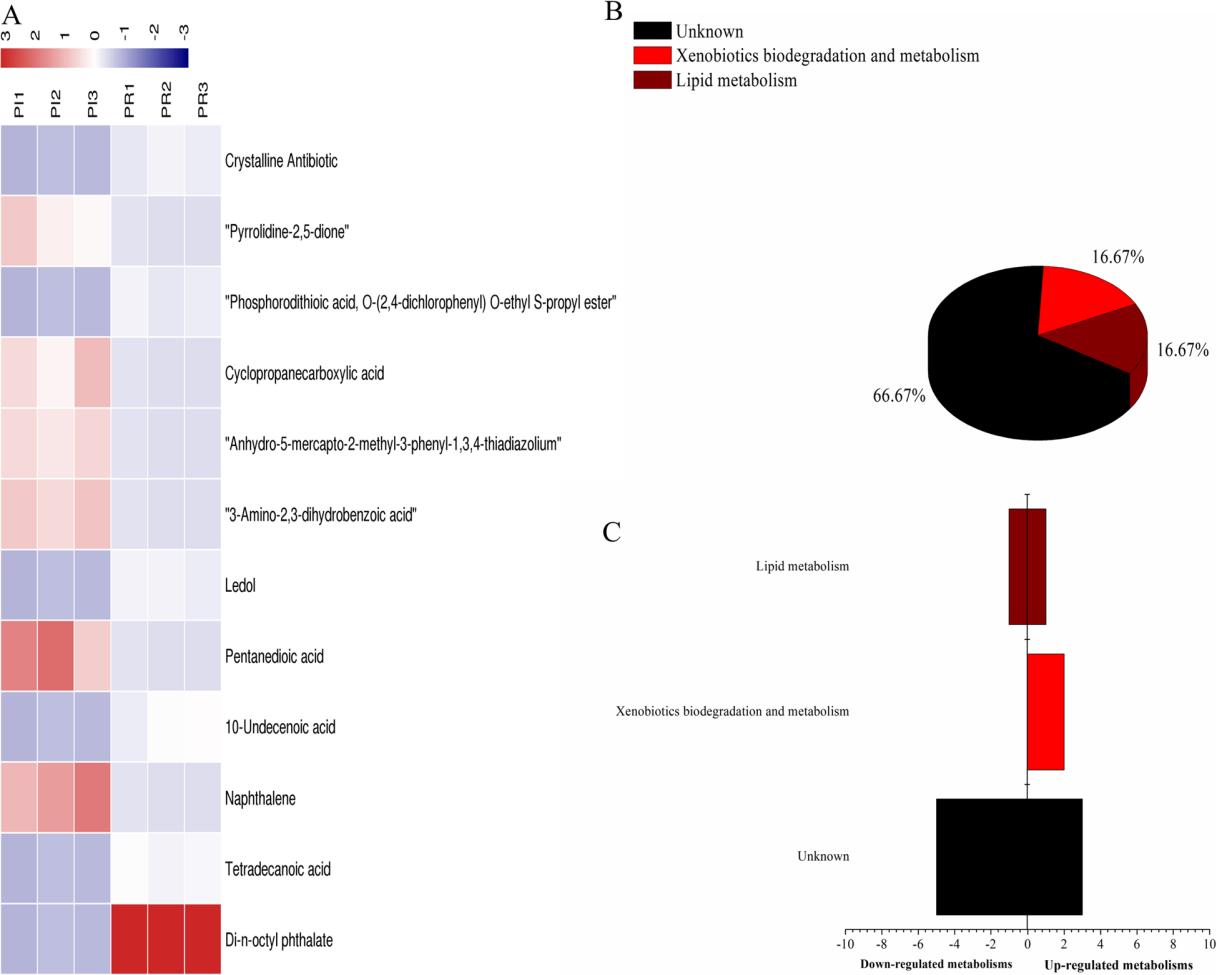
Analysis of differential metabolites between PI and PR samples absorbed by ADS-21 resin from rhizosphere soil. **a** The relative content of the metabolites between PI and PR is shown in the heat map, and the differential substances are arranged according to the VIP values from small to large. Each row represents a compound. The value of each compound represents the relative content directly normalized on the scale of the graph. Blue represents low content while red represents high content. **b** The possible metabolic pathway distribution of single metabolic substance between PI and PR. **c** The left side represents the number of single differentia substance in the same pathway where PI is lower than PR, and the right side is the opposite

In the comparison of the ADS21-extracted phase from PI and PR samples, 12 key differential metabolites were identified between the PI and PR samples, and di-n-octyl phthalate was most significantly different (VIP = 5.38322). Among the 12 differential metabolites, six were upregulated and six downregulated in the PI sample, and the same trend was also observed in the PR samples (Fig. 5a and b, Table 6). These metabolites may be involved in processes such as lysine degradation (glutarate involved) and aminobenzoate degradation (cyclopropane carboxylate involved).

Similarly, the metabolic pathways of each differential metabolite in PI and PR were analyzed (Fig. 5c and d). The contents of pentanedioic acid and cyclopropanecarboxylic acid were higher in PI than in PR, except for several differential metabolites of unknown function, which were mainly involved in xenobiotic biodegradation and metabolism.

**Fig. 6:**
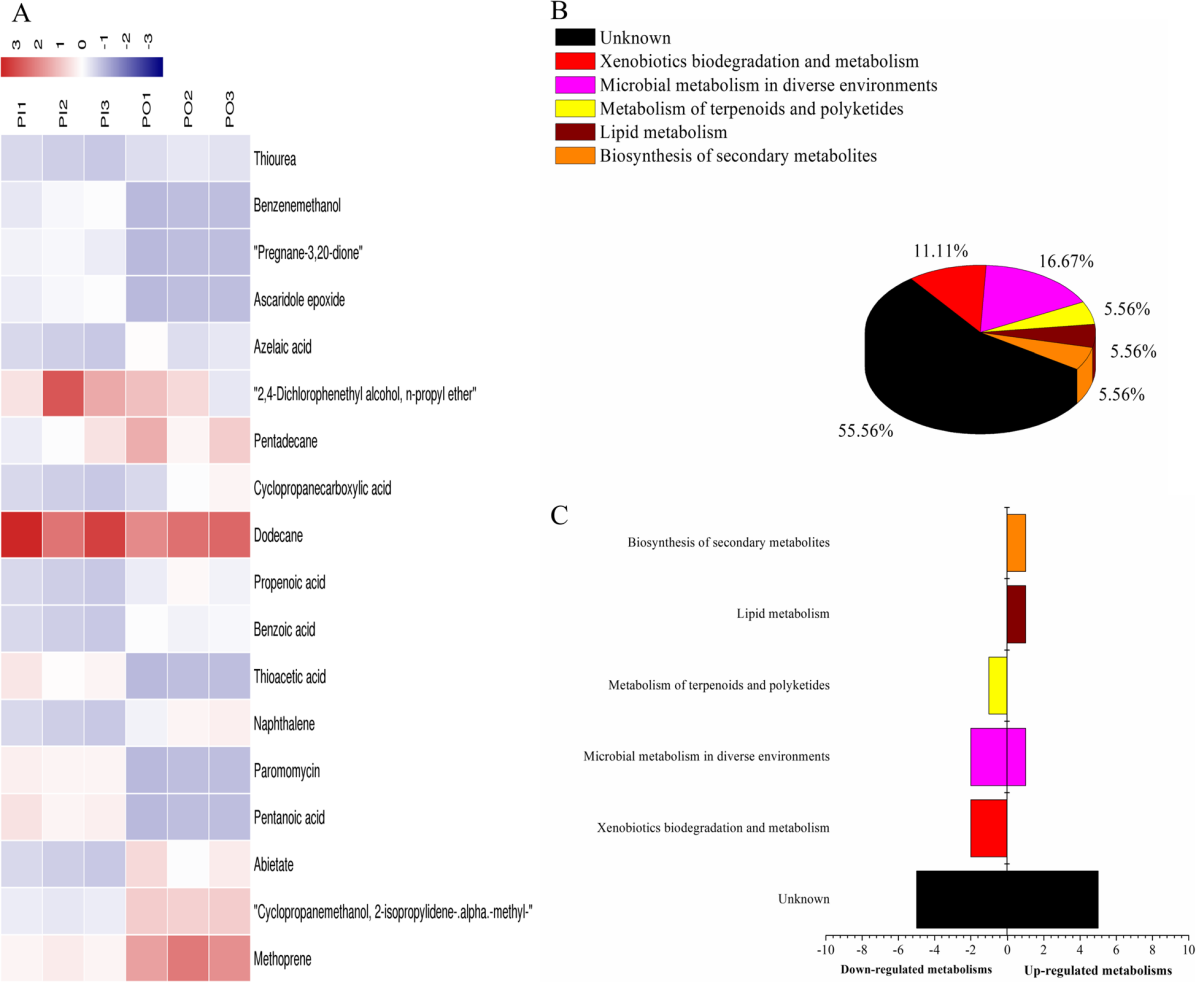
Analysis of differential metabolites between PI and PO samples absorbed by ADS-8 resin from rhizosphere soil. **a** The relative content of the metabolites between PI and PO is shown in the heat map, and the differential substances are arranged according to the VIP values from small to large. Each row represents a compound. The value of each compound represents the relative content directly normalized on the scale of the graph. Bluish color represents low content while reddish color represents high content. **b** The possible metabolic pathway distribution of single metabolic substance between PI and PO. **c** The left side represents the number of single differentia substance in the same pathway where PI is lower than PO, and the right side is the opposite

**Fig. 7:**
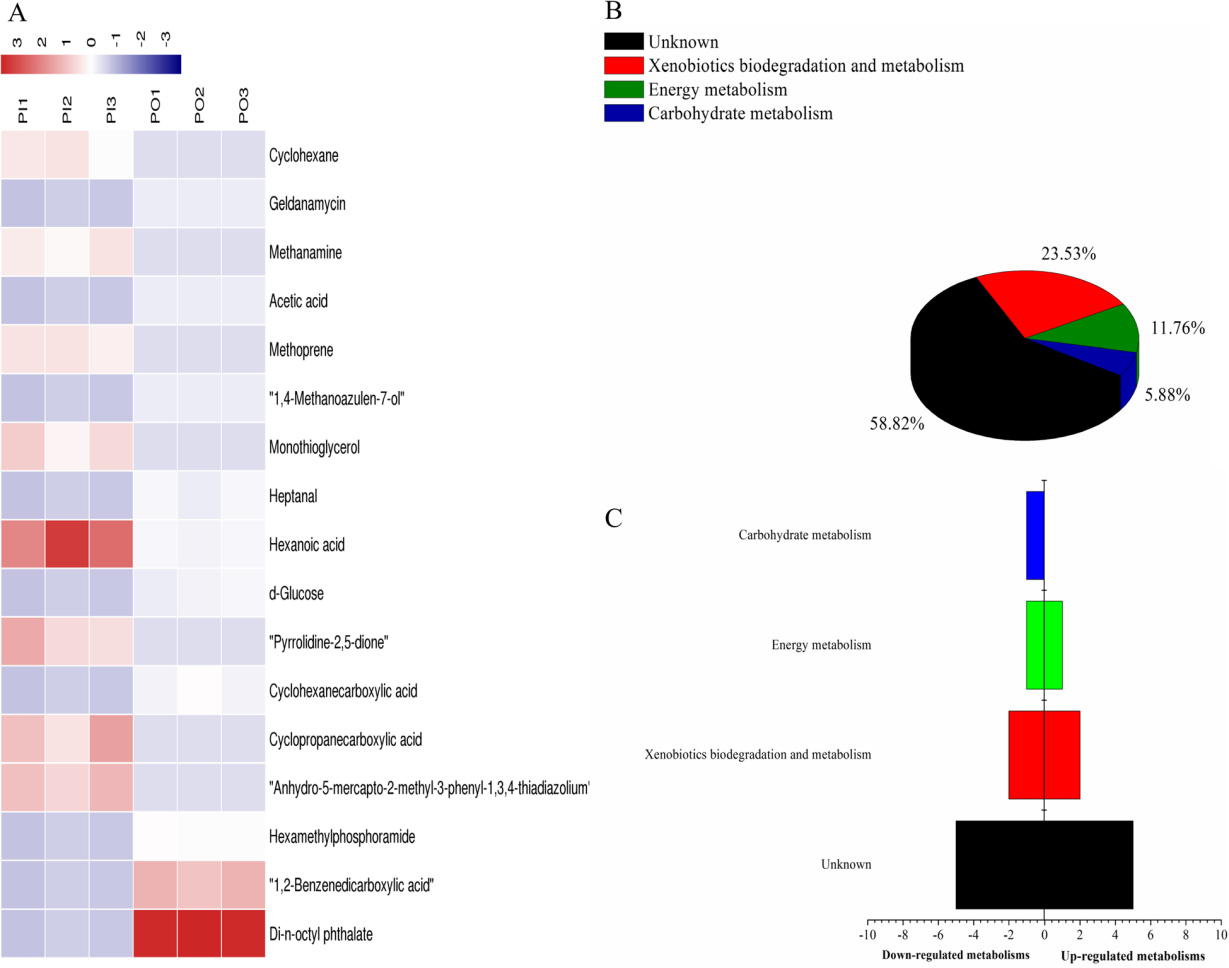
Analysis of differential metabolites between PI and PO samples absorbed by ADS-21 resin from rhizosphere soil. **a** The relative content of the metabolites between PI and PO is shown in the heat map, and the differential substances are arranged according to the VIP values from small to large. Each row represents a compound. The value of each compound represents the relative content directly normalized on the scale of the graph. Blue represents low content while red indicates high content. **b** The possible metabolic pathway distribution of single metabolic substance between PI and PO. **c** The left side represents the number of single differentia substance in the same pathway where PI is lower than PO, and the right side is the opposite

OPLS-DA analysis identified 18 and 17 differential metabolites in the 2 rhizosphere soils of the isogenic lines PI and PO by ADS8 and ADS21, respectively. The higher VIP value was methoprene (VIP = 1.85117) in ADS8 and di-n-octyl phthalate (VIP = 4.48732) in ADS21. The metabolites that were extracted by both resins possibly participate in microbial metabolism in diverse environments. At the same time, the non-polar substances extracted by the ADS8 resin may also participate in steroid hormone biosynthesis and biosynthesis of antibiotics, whereas the polar substances extracted by ADS21 may participate in neomycin, kanamycin, and gentamicin biosynthesis (Figs. 6 and 7, Tables 7 and 8).

**Table 6:** The biomarkers between PI and PR samples absorbed by ADS-21 resin in rhizosphere soil

Name	KEGG	VIP	PI	PR	Participating in metabolic pathways
Di-n-octyl phthalate	C14227	5.38322	D	U	ko01100 Metabolic pathways (2) cpd:C00829 Naphthalene cpd:C06424 Tetradecanoic acid
Tetradecanoic acid	C06424	1.55034	D	U	
Naphthalene	C00829	1.50684	U	D	
10-Undecenoic acid	C13910	1.50193	D	U	ko00310 Lysine degradation (1) cpd:C00489 Glutarate
Pentanedioic acid	C00489	1.48364	U	D	
Ledol	C09698	1.26306	D	U	ko00627 Aminobenzoate degradation (1) cpd:C16267 Cyclopropanecarboxylate
3-Amino-2,3-dihydrobenzoic acid	C12110	1.25025	U	D	
Anhydro-5-mercapto-2-methyl-3-phenyl-1,3,4-thiadiazolium	C08159	1.15087	U	D	
Cyclopropanecarboxylic acid	C16267	1.14989	U	D	
Phosphorodithioic acid, O-(2,4-dichlorophenyl) O-ethyl S-propyl ester	C18405	1.11633	D	U	
Pyrrolidine-2,5-dione	C07273	1.081	U	D	
Crystalline Antibiotic	C15751	1.06066	D	U	

Note: *U* upregulated expression. *D* downregulated expression

**Table 7:** The biomarkers between PI and PO samples absorbed by ADS-8 resin in rhizosphere soils

Name	KEGG	VIP	PI	PO	Participating in metabolic pathways
Methoprene	C14308	1.85117	D	U	ko01120 Microbial metabolism in diverse environments (5) cpd:C00180 Benzoate cpd:C00511 Acrylic acid cpd:C00556 Benzyl alcohol cpd:C00829 Naphthalene cpd:C16267 Cyclopropanecarboxylate
Cyclopropanemethanol, 2-isopropylidene-.alpha.-methyl-	C08159	1.55323	D	U	
Abietate	C06087	1.49409	D	U	
Pentanoic acid	C00803	1.47045	U	D	
Paromomycin	C00832	1.39737	U	D	
Naphthalene	C00829	1.3827	D	U	
Thioacetic acid	C01857	1.37916	U	D	ko00140 Steroid hormone biosynthesis (1) cpd:C03681 5alpha-Pregnane-3,20-dione
Benzoic acid	C00180	1.25548	D	U	
Propenoic acid	C00511	1.25174	D	U	ko01130 Biosynthesis of antibiotics (1) cpd:C00832 Paromomycin
Dodecane	C08374	1.21753	U	D	
Cyclopropanecarboxylic acid	C16267	1.18536	D	U	
Pentadecane	C08388	1.12324	D	U	
2,4-Dichlorophenethyl alcohol, n-propyl ether	C13240	1.11501	U	D	
Azelaic acid	C08261	1.09064	D	U	
Ascaridole epoxide	C09836	1.05321	U	D	
Pregnane-3,20-dione	C03681	1.02972	U	D	
Benzenemethanol	C00556	1.02626	U	D	
Thiourea	C14415	1.01138	D	U	

Note: *U* upregulated expression. *D* downregulated expression

**Table 8:** The biomarkers between PI and PO samples absorbed by ADS-21 resin in rhizosphere soil

Name	KEGG	VIP	PI	PO	Participating in metabolic pathways
Di-n-octyl phthalate	C14227	4.48732	D	U	ko01120 Microbial metabolism in diverse environments (6) cpd:C00033 Acetate cpd:C00218 Methylamine cpd:C01606 Phthalate cpd:C09822 Cyclohexane-1-carboxylate cpd:C11249 Cyclohexane cpd:C16267 Cyclopropanecarboxylate
1,2-Benzenedicarboxylic acid	C01606	2.85069	D	U	
Hexamethylphosphoramide	C19250	1.50382	D	U	
Anhydro-5-mercapto-2-methyl-3-phenyl-1,3,4-thiadiazolium	C08159	1.33164	U	D	
Cyclopropanecarboxylic acid	C16267	1.33051	U	D	
Cyclohexanecarboxylic acid	C09822	1.28803	D	U	
Pyrrolidine-2,5-dione	C07273	1.2508	U	D	
d-Glucose	C00031	1.22743	D	U	ko00524 Neomycin, kanamycin and gentamicin biosynthesis (1) cpd:C00031 D-Glucose
Hexanoic acid	C01585	1.17727	D	U	
Heptanal	C14390	1.16578	D	U	
Monothioglycerol	D05075	1.12844	U	D	
1,4-Methanoazulen-7-ol	C09631	1.08899	D	U	
Methoprene	C14308	1.0807	U	D	
Acetic acid	C00033	1.03811	D	U	
Methanamine	C00218	1.03708	U	D	
Geldanamycin	C11222	1.02144	D	U	
Cyclohexane	C11249	1.00451	U	D	

Note: *U* upregulated expression. *D* downregulated expression

The relative content of 18 kinds of differential metabolites between PI and PO and the possible metabolic pathway with a single substance involved were analyzed (Fig. 6c and d), and the results showed that the levels of pentanoic acid, paromomycin, and thioacetic acid in PI were higher than those in PO. There were more differential metabolites involved in microbial metabolism in diverse environments and xenobiotics biodegradation and metabolism, and these were down-regulated in PI. We compared with the metabolic pathways in which each differential metabolite extract by ADS21 resin might be involved in PI and PO (Fig. 7c and d) indicated that the content of d-glucose in PI was lower than that in PO. D-glucose is mainly involved in carbohydrate metabolism. Other differential metabolites and their possible metabolic pathways in PI and PO showed a similar trend.

Compared with the metabolites extracted by ADS8 of allelopathic rice PI and its transgenic rice PR and PO, we found that the unique metabolites of PR were paromomycin and benzamide, whereas that of PO were naphthalene and thiourea. When the *PAL* gene was overexpressed, the levels of seven substances decreased, which included dodecane, cucurbitacin b, and butane, whereas those of cyclopropanemethanol, 2-isopropylidene-.alpha.-methyl-, acetic acid, and pentadecane increased. At the same time, after *PAL* gene interference, the levels of five substances decreased (including cyclohexanone, paromomycin, pentanoic acid, thioacetic acid, and preananne-3,20-dione), whereas those of ascaridole epoxide and benzenemethanol increased.

Comparison of the ADS21-extracted phase from the PI, PR, and PO samples showed that the concentrations of 8 polar metabolites decreased after interference or overexpression of the *PAL* gene, e.g., methoprene, pyrrolidine-2,5-dione, and cyclohexane. However, the concentrations of tetradecanoic acid and 10-undecenoic acid increased with interference of the *PAL* gene. However, when the *PAL* gene was overexpressed, the levels of seven metabolites increased.

### Differences in metabolites from the rhizosphere soils of Le and LO

**Fig. 8:**
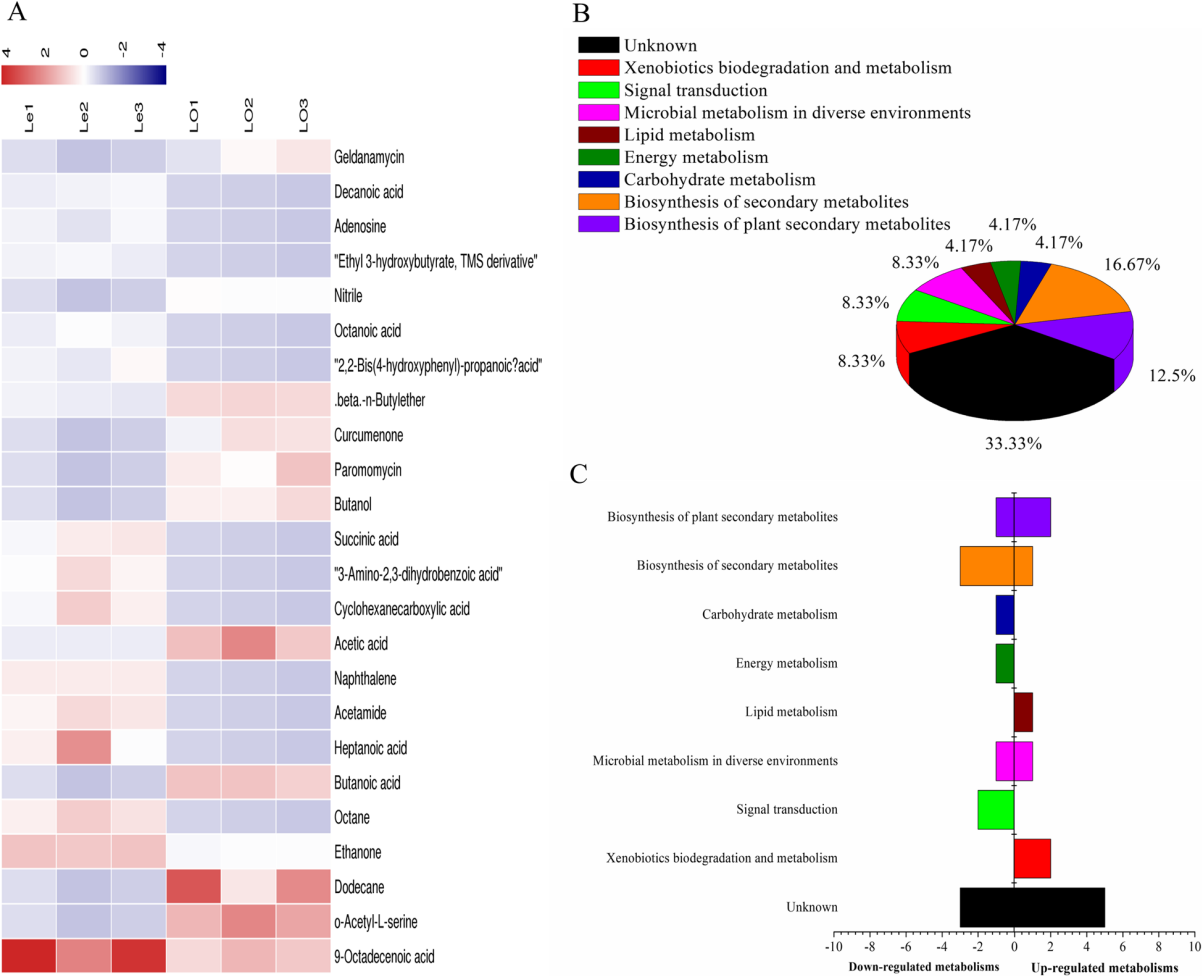
Analysis of differential metabolites between Le and LO samples absorbed by ADS-8 resin from rhizosphere soil. **a** The relative content of the metabolites between Le and LO is shown in the heat map, and the differential substances are arranged according to the VIP values from small to large. Each row represents a compound. The value of each compound represents the relative content directly normalized on the scale of the graph. Blue represents low content while red represents high content. **b** The possible metabolic pathway distribution of single metabolic substance between Le and LO. **c** The left side represents the number of single differentia substance in the same pathway where Le is lower than LO, and the right side is the opposite

Figure 8a and b and Table 9 indicated that of the differentially expressed metabolites identified, 14 were up-regulated and 10 were down-regulated in the Le samples, and a more significant differential expression was observed for metabolites 9-octadecenoic acid (VIP = 2.48717) and o-acetyl-L-serine (VIP = 2.03914). Analysis of the contents of 24 differential metabolites in Le and LO and the metabolic pathways in which single differential metabolites may be involved (Fig. 8c and d) showed that the contents of acids (such as 9-octadecanoic acid, heptanoic acid, and succinic acid), ethenone, and octane in Le were higher than in LO. The differential metabolites involved in the biosynthesis of plant secondary metabolites, xenobiotics biodegradation and metabolism, and lipid metabolism were upregulated in Le.

**Table 9:** The biomarkers between Le and LO samples absorbed by ADS-8 resin in rhizosphere soil

Name	KEGG	VIP	Le	LO	Participating in metabolic pathways
9-Octadecenoic acid	C00712	2.48717	U	D	ko01100 Metabolic pathways (9) cpd:C00033 Acetate cpd:C00042 Succinate cpd:C00084 Acetaldehyde cpd:C00152 L-Asparagine cpd:C00212 Adenosine cpd:C00829 Naphthalene cpd:C00979 O-Acetyl-L-serine cpd:C01571 Decanoic acid cpd:C06423 Octanoic acid
o-Acetyl-L-serine	C00979	2.03914	D	U	
Dodecane	C08374	1.95655	D	U	
Ethanone	C00084	1.76858	U	D	
Octane	C01387	1.7625	U	D	
Butanoic acid	C00246	1.70646	D	U	
Heptanoic acid	C17714	1.68056	U	D	
Acetamide	C06244	1.66271	U	D	
Naphthalene	C00829	1.63686	U	D	
Acetic acid	C00033	1.57475	D	U	
Cyclohexanecarboxylic acid	C09822	1.49533	U	D	ko01040 Biosynthesis of unsaturated fatty acids (1) cpd:C00712 (9Z)-Octadecenoic acid
3-Amino-2,3-dihydrobenzoic acid	C12110	1.48714	U	D	
Succinic acid	C00042	1.44648	U	D	ko00524 Neomycin, kanamycin and gentamicin biosynthesis (1) cpd:C00832 Paromomycin
Butanol	C06142	1.39642	D	U	
Paromomycin	C00832	1.36792	D	U	
Curcumenone	C17492	1.24441	D	U	
.beta.-n-Butylether	C00152	1.1672	D	U	
2,2-Bis(4-hydroxyphenyl)-propanoic acid	C13633	1.14348	U	D	
Octanoic acid	C06423	1.11278	U	D	
Nitrile	C00726	1.10806	D	U	
Ethyl 3-hydroxybutyrate, TMS derivative	C03499	1.09261	U	D	
Adenosine	C00212	1.08609	U	D	
Decanoic acid	C01571	1.08017	U	D	
Geldanamycin	C11222	1.00891	D	U	

Note: *U* upregulated expression. *D* downregulated expression

**Fig. 9:**
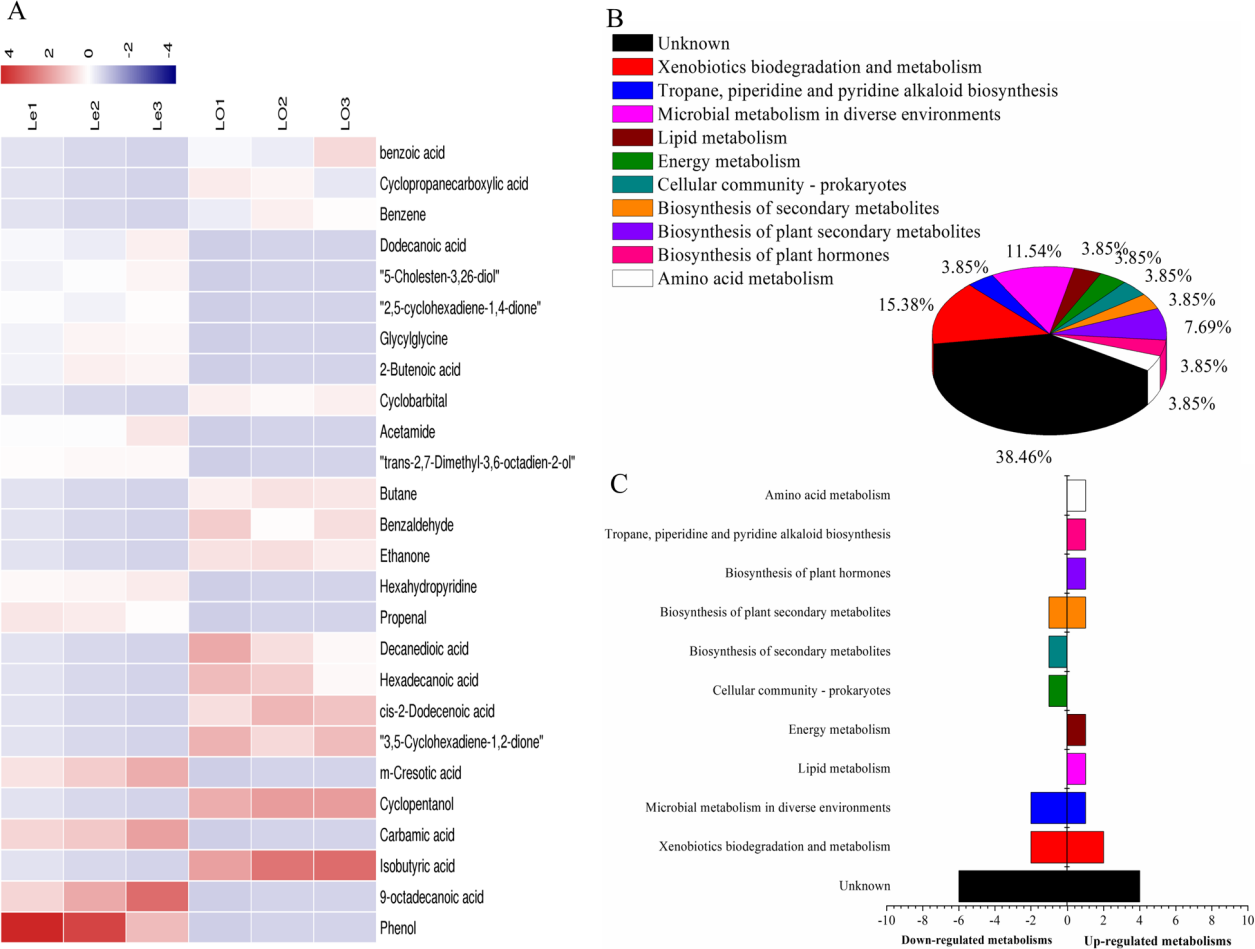
Analysis of differential metabolites between Le and LO samples absorbed by ADS-21 resin from rhizosphere soil. **a** The relative content of the metabolites between Le and LO is shown in the heat map, and the differential substances are arranged according to the VIP values from small to large. Each row represents a compound. The value of each compound represents the relative content directly normalized on the scale of the graph. Blue represents low content while red indicates high content. **b** The possible metabolic pathway distribution of single metabolic substance between Le and LO. **c** The left side represents the number of single differentia substance in the same pathway where Le is lower than LO, and the right side is the opposite

Figure 9a and b and Table 10 show that on the basis of metabolomics analysis, 26 differential metabolites were identified in the Le and LO samples, of which phenol and 9-octadecanoic acid compounds showed the most significant differences. The two metabolites were upregulated in the Le samples, but downregulated in the LO samples. Based on the analysis of the data of Le and LO (Figs. 9c and d), it was found that the content of differential metabolites involved in lipid metabolism, energy metabolism, biosynthesis of other secondary metabolites, biosynthesis of plant hormones, and amino acid metabolism in Le was higher than in LO, but the content of differential foreign bodies involved in biosynthesis of secondary metabolites and cellular community-prokaryotes was lower than in LO.

**Table 10:** The biomarkers between Le and LO samples absorbed by ADS-21 resin in rhizosphere soil

Name	KEGG	VIP	Le	LO	Participating in metabolic pathways
Phenol	C00146	2.82987	U	D	ko01120 Microbial metabolism in diverse environments (8) cpd:C00049 L-Aspartate cpd:C00084 Acetaldehydecpd:C00146 Phenolcpd:C00180 Benzoatecpd:C00472 p-Benzoquinonecpd:C01407 Benzenecpd:C02632 2-Methylpropanoatecpd:C16267 Cyclopropanecarboxylate
9-octadecanoic acid	C01530	2.27918	U	D	
Isobutyric acid	C02632	2.19198	D	U	
Carbamic acid	C01563	2.07028	U	D	
Cyclopentanol	C02020	1.96481	D	U	
m-Cresotic acid	C14103	1.94407	U	D	
3,5-Cyclohexadiene-1,2-dione	C02351	1.67809	D	U	
cis-2-Dodecenoic acid	C21202	1.6433	D	U	
Hexadecanoic acid	C00249	1.4779	D	U	
Decanedioic acid	C08277	1.43455	D	U	ko01040 Biosynthesis of unsaturated fatty acids (2) cpd:C00249 Hexadecanoic acid cpd:C01530 Octadecanoic acid
Propenal	C01471	1.43094	U	D	
Hexahydropyridine	C01746	1.41484	U	D	
Ethanone	C00084	1.39731	D	U	ko00982 Drug metabolism - cytochrome P450 (1) cpd:C01471 Acrolein
Benzaldehyde	C00193	1.36669	D	U	
Butane	D03186	1.344	D	U	ko00240 Pyrimidine metabolism (1) cpd:C01563 Carbamate
Purine-2-acetamide, trans-2,7-Dimethyl-3,6-octadien-2-ol	C01500	1.29879	U	D	
Acetamide	C06244	1.27783	U	D	ko00960 Tropane, piperidine and pyridine alkaloid biosynthesis (1) cpd:C01746 Piperidine
Cyclobarbital	D07323	1.22403	D	U	
2-Butenoic acid	C01771	1.20318	U	D	ko00120 Primary bile acid biosynthesis (1) cpd:C17336 7alpha,26-Dihydroxy-4-cholesten-3-one
Glycylglycine	C02037	1.16897	U	D	
2,5-cyclohexadiene-1,4-dione	C00472	1.12428	U	D	
5-Cholesten-3,26-diol	C17336	1.10082	U	D	
Dodecanoic acid	C02679	1.09957	U	D	
Benzene	C01407	1.05894	D	U	
Cyclopropanecarboxylic acid	C16267	1.05361	D	U	
Benzoic acid	C00180	1.04824	D	U	

Note: *U* upregulated expression. *D* downregulated expression

Comparison of possible pathways of metabolites between the two resins extracted showed that the substances extracted by ADS8 were more likely involved in the four pathways (biosynthesis of plant secondary metabolites, biosynthesis of secondary metabolites, energy metabolism, and lipid metabolism) than in ADS21, whereas the involvement of the two pathways (microbial metabolism in diverse environments and xenobiotics biodegradation and metabolism) decreased.

## Discussion

Previous studies have shown that plants gather specific microbial communities and form unique rhizosphere microbial community structure through the mediation of root exudates [28–30]. The unique microecological environment is composed of plants, root exudates, and root microorganisms, which cause allelopathy [36–42]. Previous studies have demonstrated that the community and population of bacteria, fungi, and actinomycetes significantly differed in the rhizosphere soil of allelopathic and non-allelopathic rice under the mediation of different exudates [22, 32]. Accordingly, phenolic acids and terpenes could specifically aggregate certain microorganisms [43, 44]. Most of the existing studies have focused on the differences and functions of phenolic acids and terpenes, as well as their relationship with microorganisms in rice. Limited research has referred to other potential allelopathic substances, such as aromatic acid, benzene, derivatives, long-chain hydrocarbons, fatty acids, etc. [45].

Among these compounds, some long-chain fatty acids are known phytotoxins [1]. For example, nonanoic and decanoic acids have phytotoxic effects on algae as indicated by bioassays [46], and pelargonic acid (nonanoic acid) is regarded as a natural herbicide based on its particular phytotoxicity [47].

In the present study, the concentrations of several fatty acids varied among allelopathic potential rice lines. The resin adsorbed phase extracted from the rhizosphere soil of PI and Le at the five-leaf stage showed that the metabolites extracted from different allelopathic potential rice accessions were significantly different. The metabolites of allelopathic rice PI and non-allelopathic rice Le were analyzed and indicated that 21 different metabolites were extracted from rice rhizosphere soil by ADS-8 resin, of which acetic acid was the most significantly differentially expressed metabolite. The rhizosphere soil of allelopathic rice PI contained more acetic acid than non-allelopathic rice Le. Acetic acid is a weak carboxylic acid; in wheat, the anaerobic decomposition of wheat straw released acetic acid and this compound exhibited phytotoxicity [48].

The relative content of acetic acid was higher in the high allelopathic rice than the low allelopathic rice, which may be associated with rice allelopathy, and the compound could be generated from the anaerobic reaction and play a role in inhibiting weed growth. It is also regarded as a precursor in the process of biosynthesizing phenolic acids, terpenes and other metabolites [49].

In addition, long-chain fatty acids have also been documented as allelochemicals, with maize straw decomposed products showing allelopathic promotion or inhibition of the soil-born microorganisms, and several fatty acids were identified from these decomposed compounds, which included hexanoic acid (1.73%), 8-octadecenoic acid (1.06%), and 3-(4-hydroxy-3-methoxy-phenyl)-2-propenoic acid (1.04%) [50]. The content of fatty acids were differed in rhizosphere soil of PI and Le, which would lead to the variations in allelopathic inhibition activity on weeds. Since PI and Le have different genetic backgrounds, when it turns to a same rice line with *PAL* overexpressed or silenced, the fatty acids from these transformed rice lines also differed to the WT, which indicated that changes in gene expression of *PAL* in rice results in changes in fatty acid contents in their rhizosphere and suggesting a putative link between *PAL* expression and fatty acids. The fatty acids would impact on the soil environment, since hexadecanoic acid from the root exudates of peanut (*Arachis hypogaea* L.) has close relationship with its soil sickness, high concentration (160 mg/kg soil and 240 mg/kg soil) of hexadecanoic acid would suppress the soil enzyme activity, which in turn to reduce root activity and the chlorophyll content in peanut leaves [51]. The hexadecanoic acid in the rhizosphere soil of PI and LO extracted by ADS21 resin was higher than that in Le. Fischer et al. also believed that long-chain fatty acids may interact with plant lipids (sterols etc.) to form micelle as allelochemicals [52]. Therefore, it was speculated that long-chain fatty acids act roles in directly impact on the soil microorganism, or interacting with other metabolites to form mixture with alleloapthic activity.

In terms of other metabolites, except for fatty acids, many are also involved in microbial or plant metabolism. Phenol, for example, is the metabolic substrate of various microorganisms, such as Pseudomonas [53], Acinetobacter [54], and Rhodococcus [55]. Wild watermelon and *Cucurbita maxima* could produce cucurbitacin b, which could inhibit cancer cells and Meloidogyne species population densities [56, 57]. *Glomerella cingulata* could transform ledol [58]. The differences in these substances may lead to different allelopathic potentials in rice. However, the relationship between these substances and rice allelopathy requires further investigation.

However, the exact role of long-chain fatty acids and acetic acid on the diversities of microbial community in the rhizosphere and their correlation with weed suppression still needs in-depth study.

## Conclusions

Our study showed that the rhizosphere soil of allelopathic rice accumulates more non-polar metabolites than polar metabolites, and fatty acids are regarded as vital compounds, which is different from the well-known allelochemicals, including phenolic acids and terpenoids. The fatty acids might be indispensable for the activity of rhizospheric microorganisms, which helps in constructing the micro-environment to assist in maintaining allelopathic activity.

## Methods

### Plant growth conditions

This experiment was conducted in 2016 at Fujian Agriculture and Forestry University, Fuzhou, China, where temperature varied from of 25 °C to 30 °C, while humidity varied from 65 to 78% during the experiment. The site is the outdoor network room experimental field.

The globally known PI312777, the stronger allelopathic rice (PI, introduced from the USA), its transgenic lines with *PAL2–1*overexpression (PO) and with *PAL2–1* interfered expression (PR), non-allelopathic rice Lemont (Le, introduced from the USA), and its transgenic line with *PAL2–1* overexpression (LO) were used for this study. All of the tested materials were genetically stable ones selected for multiple generations provided by Institute of Agroecology, Fujian Agriculture and Forestry University.

The dry-raised seedlings were transplanted following the method as described by Li et al. [59]. The field soil was sandy loam, and there was 1.72 g·kg^− 1^ total nitrogen, 60.67 mg·kg^− 1^ alkali hydrolysable nitrogen, 0.67 g·kg^− 1^total phosphorus, 30.45 mg·kg^− 1^ available phosphorus, 1.53 g·kg^− 1^ total potassium, 206.48 mg·kg^− 1^ available potassium, and 21.3 g·kg^− 1^ organic matter, with pH 6.13 in the tillage layer. The growth of rice seedlings at seedling stage was observed in random plants, and the soil sampling was taken at the five-leaf stage.

Rice seedlings were transplanted on March 25, 2016 with appropriate basal fertilizers (70% of total N as basal dressing and top dressing, in total *N* = 225 kg/hm^2^, N: P: K = 1:0.5:0.8). Each variety was planted separately in different plots (3 m × 1 m) with a seeding rate of 150 g/m^2^. In addition, CK was set as the blank control soil in the same area without rice planting. After the emergence of rice seedlings, the recommended field management practices were followed.

### Soil sample collection

Random sampling was conducted in field at the five-leaf stage of rice seedlings. The sample collection method was followed by the method with slightly improvement [60]. Briefly, the rhizospheric soil was collected by shaking the roots to remove soil lumps sticking to the roots, and the remaining soil clinging to the roots was removed with forceps and put into a 50 mL centrifuge tube. Then 50 mL of PBS solution (pH = 7.0) was added and ultrasonically oscillated for 10 min. Subsequently, the plant materials were removed from the resulting solution after centrifugation. Lastly, the control samples were collected from uncultivated soil at the depth of 0–5 cm.

### Extraction and identification of metabolites from rhizosphere soils of different rice accessions

Six samples were taken from the rhizosphere soil of each treatment. Ten grams of each sample was added with 30 mL of sterile water, and oscillated at 200 rpm at 20°C for 3 h, then centrifuged three times at 12,000×g, for 10 min. Finally, the supernatants were pooled, mixed well, and divided evenly into two portions.

The resin ADS-8 (weakly polar resin) and ADS-21 (polar resin) (purchased from Tianjin Nankai Hecheng Co., Ltd.), respectively, were weighed with 100 g each. After pre-treatment, these were added to the above water extracts at 20 °C and then oscillated at 200 rpm for 24 h. Later, the resins were removed, 100 mL of methanol was added for elution, and then oscillated at 200 rpm at 20 °C for 2 h with three repeats, then the methanol phase was combined and vacuum concentrated to dry. Then, 1 mL of methanol was added to dissolve resin, filtered across a 0.22 mm mixed cellulose membrane, and then the metabolites were analyzed.

A Shimadzu GCMS-TQ8040 Gas Chromatography/Triple four-stage bar mass spectrometer was used to analyze the above processed samples using the following conditions: The initial temperature was set at 60 °C for 2 min, then it was increased to 120 °C at 8 °C/min for 2 min, and then further increased to 210 °C at 10 °C/min for 3 min. Finally, the temperature rose to 250 °C at 15 °C /min for 5 min. Injection port temperature was 250 °C. Carrier gas: He, Flow: 1 mL·min^-1^; Injection volume: 1 μL. Mass spectrum: electron bombardment of ion sources (EI). The samples were scanned at 40–650 amu.

## Supplementary information

**Additional file 1: Figure S1.** The original full-length Western-blot images. A: Western blotting detection of the *Os*PAL2–1 expression on rice roots. B: Western blotting detection of the β-actin expression on rice roots.

**Additional file 2: Figure S2.** Principal component analysis (PCA) is a reliable method for simplifying and reducing multidimensional data in an unsupervised pattern. On the basis of preserving the original data to the greatest extent, a linear combination was performed to determine the trend of metabolome separation between the samples of each group. PCA of the compounds in absorption phase by ADS-8 resin from rhizosphere soils of different allelopathic potential rice accessions. Note: The numbers 1, 2, and 3 in the figure represent three parallel duplicates of the same sample. PI stands for allelopathic rice PI312227 (PI). PAL for *PAL2–1* inhibited transgenic line PR. O for the transgenic line PO of *PAL2–1* overexpressed in allelopathic rice PI312227 (PI). Le for non-allelopathic rice Lemont (Le). LOP for the transgenic line LO of *PAL2–1* overexpressed in non-allelopathic rice Le.

**Additional file 3: Figure S3.** Principal component analysis of the compounds absorbed by ADS-21 resin from the rhizosphere soils of different potential allelopathic rice accessions. Note: The numbers 1, 2, and 3 in the figure represent three parallel duplicates of the same sample. PI stands for allelopathic rice PI312227 (PI). PAL for *PAL2–1* inhibited transgenic line PR. O for the transgenic line PO of *PAL2–1* overexpressed in allelopathic rice PI312227 (PI). Le for non-allelopathic rice Lemont (Le). LOP for the transgenic line LO of *PAL2–1* overexpressed in non-allelopathic rice Le.

**Additional file 4: Figure S4.** Metabolomics analysis was conducted to identify the soil substances in the extracted phase by ADS-8 resin from the rhizosphere soils of different allelopathic potential rice accessions and OPLS-DA evaluation model was established. The OPLS-DA score of the compounds in the absorption phase by ADS-8 resin from the rhizosphere soils of different allelopathic potential rice accessions. Note: The numbers 1, 2, and 3 in the figure represent three parallel duplicates of the same sample. PI stands for allelopathic rice PI312227 (PI). PAL for *PAL2–1* inhibited transgenic line PR. O for the transgenic line PO of *PAL2–1* overexpressed in allelopathic rice PI312227 (PI). Le for non-allelopathic rice Lemont (Le). LOP for the transgenic line LO of *PAL2–1* overexpressed in non-allelopathic rice Le. Picture order from top to next: PI-Le, PI-PR, PI-PO, Le-LO, PO-LO.

**Additional file 5: Figure S5.** The OPLS-DA score of the compounds absorbed by ADS-21 resin from the rhizosphere soils of different allelopathic potential rice accessions. It shows that the predictive parameters of the OPLS-DA model, R2X, R2Y and Q2, were both greater than 0.9, suggesting that the model is excellent and suitable for further analysis of differential metabolites. Note: The numbers 1, 2, and 3 in the figure represent three parallel duplicates of the same sample. PI stands for allelopathic rice PI312227 (PI). PAL for *PAL2–1* inhibited transgenic line PR. O for the transgenic line PO of *PAL2–1* overexpressed in allelopathic rice PI312227 (PI). Le for non-allelopathic rice Lemont (Le). LOP for the transgenic line LO of *PAL2–1* overexpressed in non-allelopathic rice Le. Picture order from top to next: PI-Le, PI-PR, PI-PO, Le-LO, PO-LO.

## Data Availability

All data generated during this study are included in this published article and its supplementary information files, and the raw data used or analysed during the current study available from the corresponding author on reasonable request.

## Abbreviations

*PAL*: Phenylalanine ammonia-lyase gene
*PAL2–1*: Phenylalanine ammonia-lyase (*PAL*) gene 2–1
WT: Wild-type
PI: PI312777, the stronger allelopathic rice
PR: Transgenic line with major allelopathic gene *PAL2–1*-inhibited of PI
PO: Transgenic line with *PAL2–1*overexpression of PI
Le: Non-allelopathic rice Lemont
LO: Transgenic line with *PAL2–1* overexpression of Le
CK: The blank control soil
KEGG: Kyoto Encyclopedia of Genes and Genomes
PBS solution: Phosphate buffer saline
PCA: Principal Component Analysis
OPLS-DA: Orthogonal partial least squares discriminant analysis
VIP: Projection of variable importance of OPLS-DA model
