# Supplementary material for: Metabolite profiling of rhizosphere soil of different allelopathic potential rice accessions
Source: BMC Plant Biol. 2020 Jun 9;20:265. doi: 10.1186/s12870-020-02465-6 (PMC7282037; doi:10.1186/s12870-020-02465-6)
Supplement: Supplementary file 1 — Additional file 1: Figure S1. The original full-length Western-blot images. A: Western blotting detection of the OsPAL2–1 expression on rice roots. B: Western blotting detection of the β-actin expression on rice roots. [file 12870_2020_2465_MOESM1_ESM.docx]

**
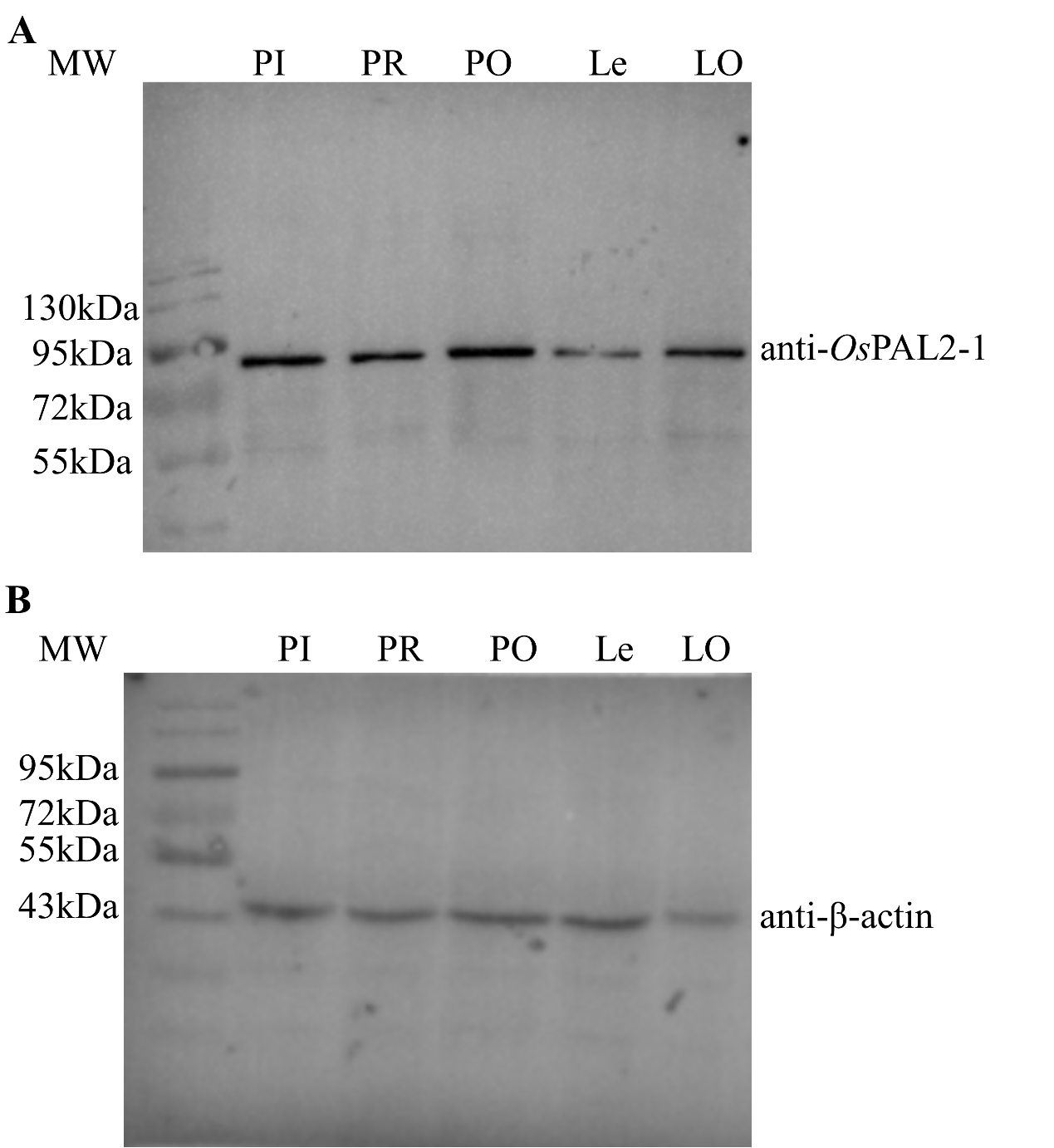
**

**Additional file 1: Figure S1.** The original full-length western-blot images. A: Western blotting detection of the OsPAL2-1 expression on rice root. B: Western blotting detection of the β-actin expression on rice root.
