# Supplementary material for: Metabolite profiling of rhizosphere soil of different allelopathic potential rice accessions
Source: BMC Plant Biol. 2020 Jun 9;20:265. doi: 10.1186/s12870-020-02465-6 (PMC7282037; doi:10.1186/s12870-020-02465-6)
Supplement: Supplementary file 2 — Additional file 2: Figure S2. Principal component analysis (PCA) is a reliable method for simplifying and reducing multidimensional data in an unsupervised pattern. On the basis of preserving the original data to the greatest extent, a linear combination was performed to determine the trend of metabolome separation between the samples of each group. PCA of the compounds in absorption phase by ADS-8 resin from rhizosphere soils of different allelopathic potential rice accessions. Note: The numbers 1, 2, and 3 in the figure represent three parallel duplicates of the same sample. PI stands for allelopathic rice PI312227 (PI). PAL for PAL2–1 inhibited transgenic line PR. O for the transgenic line PO of PAL2–1 overexpressed in allelopathic rice PI312227 (PI). Le for non-allelopathic rice Lemont (Le). LOP for the transgenic line LO of PAL2–1 overexpressed in non-allelopathic rice Le. [file 12870_2020_2465_MOESM2_ESM.docx]

**
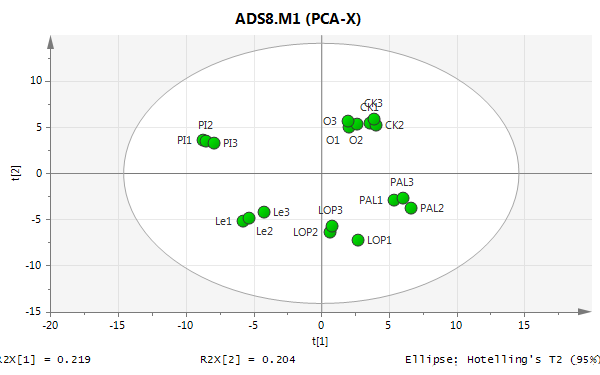
**

**Additional file 2: Figure S2.** Principal component analysis (PCA) is a reliable method for simplifying and reducing multidimensional data in an unsupervised pattern. On the basis of preserving the original data to the greatest extent, a linear combination was performed to determine the trend of metabolome separation between the samples of each group. PCA of the compounds in absorption phase by ADS-8 resin from rhizosphere soils of different allelopathic potential rice accessions. Note: The numbers 1,2, and 3 in the figure represent three parallel duplicates of the same sample. PI stands for allelopathic rice PI312227 (PI). PAL for PAL2-1 inhibited transgenic line PR. O for the transgenic line PO of PAL2-1 overexpressed in allelopathic rice PI312227 (PI). Le for non-allelopathic rice Lemont (Le). LOP for the transgenic line LO of PAL2-1 overexpressed in non-allelopathic rice Le.
