# Supplementary material for: Metabolite profiling of rhizosphere soil of different allelopathic potential rice accessions
Source: BMC Plant Biol. 2020 Jun 9;20:265. doi: 10.1186/s12870-020-02465-6 (PMC7282037; doi:10.1186/s12870-020-02465-6)
Supplement: Supplementary file 5 — Additional file 5: Figure S5. The OPLS-DA score of the compounds absorbed by ADS-21 resin from the rhizosphere soils of different allelopathic potential rice accessions. It shows that the predictive parameters of the OPLS-DA model, R2X, R2Y and Q2, were both greater than 0.9, suggesting that the model is excellent and suitable for further analysis of differential metabolites. Note: The numbers 1, 2, and 3 in the figure represent three parallel duplicates of the same sample. PI stands for allelopathic rice PI312227 (PI). PAL for PAL2–1 inhibited transgenic line PR. O for the transgenic line PO of PAL2–1 overexpressed in allelopathic rice PI312227 (PI). Le for non-allelopathic rice Lemont (Le). LOP for the transgenic line LO of PAL2–1 overexpressed in non-allelopathic rice Le. Picture order from top to next: PI-Le, PI-PR, PI-PO, Le-LO, PO-LO. [file 12870_2020_2465_MOESM5_ESM.docx]

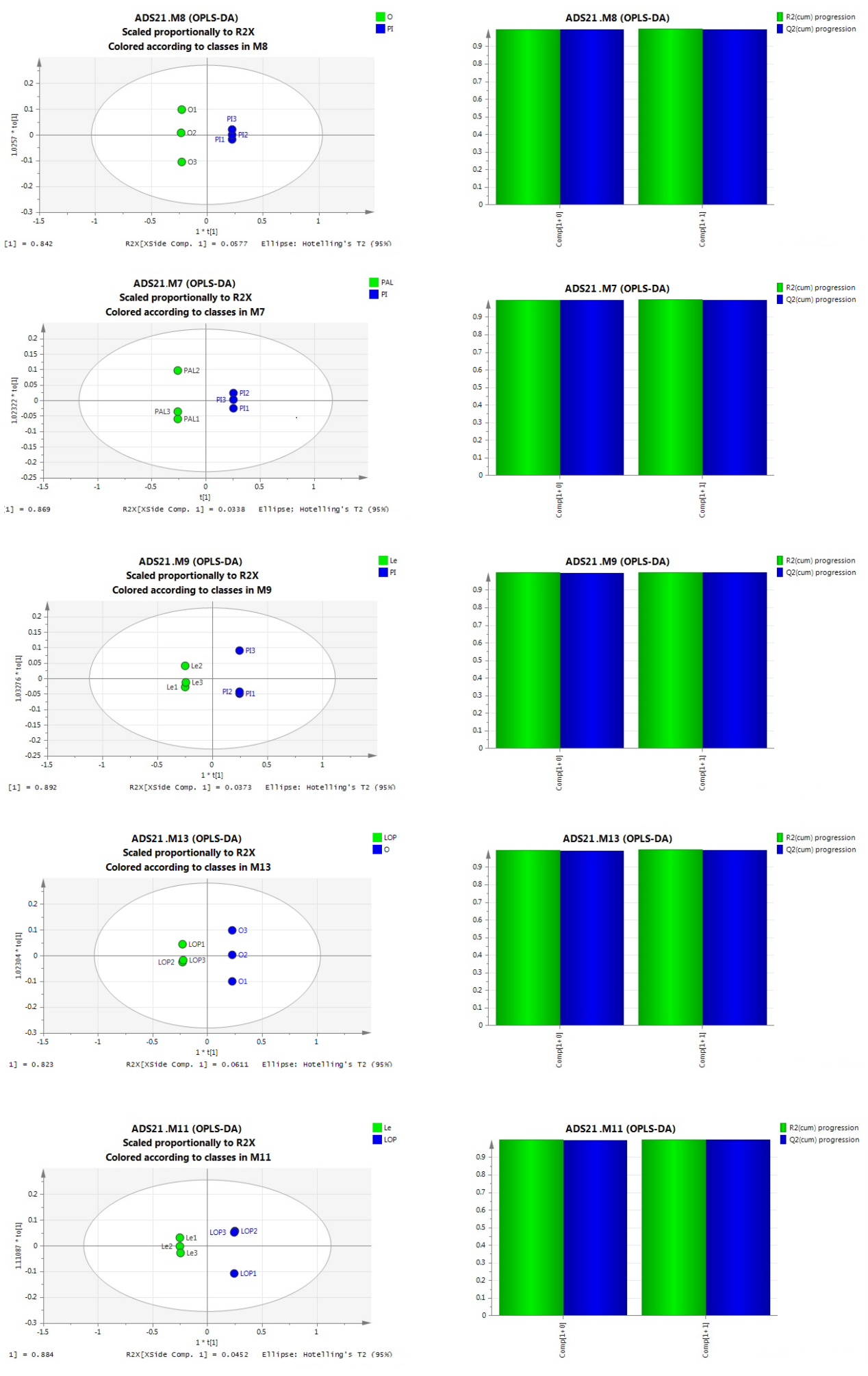


**Additional file 5: Figure S5.** The OPLS-DA score of the compounds absorbed by ADS-21 resin from the rhizosphere soils of different allelopathic potential rice accessions. It shows that the predictive parameters of the OPLS-DA model, R2X, R2Y and Q2, were all greater than 0.9, suggesting that the model is excellent and could therefore be used for further analysis of differential metabolites. Note: The numbers 1, 2, and 3 in the figure represent three parallel duplicates of the same sample. PI stands for allelopathic rice PI312227 (PI). PAL for PAL2-1 inhibited transgenic line PR. O for the transgenic line PO of PAL2-1 overexpressed in allelopathic rice PI312227 (PI). Le for non-allelopathic rice Lemont (Le). LOP for the transgenic line LO of PAL2-1 overexpressed in non-allelopathic rice Le. Picture order from top to next: PI-Le, PI-PR, PI-PO, Le-LO, PO-LO.
